# Beyond controlling cell size: functional analyses of S6K in tumorigenesis

**DOI:** 10.1038/s41419-022-05081-4

**Published:** 2022-07-25

**Authors:** Xueji Wu, Wei Xie, Wenxuan Xie, Wenyi Wei, Jianping Guo

**Affiliations:** 1 Department of Liver Surgery, Hepatobiliary Pancreatic Center, the First Affiliated Hospital, Sun Yat-sen University, Guangzhou, Guangdong 5100080 China; 2 Institute of Precision Medicine, the First Affiliated Hospital, Sun Yat-sen University, Guangzhou, Guangdong 5100080 China; 3 Department of Pathology, Beth Israel Deaconess Medical Center, Harvard Medical School, Boston, MA 02115 USA

**Keywords:** Oncogenes, Insulin signalling

## Abstract

As a substrate and major effector of the mammalian target of rapamycin complex 1 (mTORC1), the biological functions of ribosomal protein S6 kinase (S6K) have been canonically assigned for cell size control by facilitating mRNA transcription, splicing, and protein synthesis. However, accumulating evidence implies that diverse stimuli and upstream regulators modulate S6K kinase activity, leading to the activation of a plethora of downstream substrates for distinct pathobiological functions. Beyond controlling cell size, S6K simultaneously plays crucial roles in directing cell apoptosis, metabolism, and feedback regulation of its upstream signals. Thus, we comprehensively summarize the emerging upstream regulators, downstream substrates, mouse models, clinical relevance, and candidate inhibitors for S6K and shed light on S6K as a potential therapeutic target for cancers.

## Facts

- S6K canonically governs cell size by facilitating mRNA transcription, splicing, and protein synthesis.
- S6K also plays pivotal role in directing cell apoptosis, metabolism, and feedback regulation of its upstream signals.
- Diverse stimuli and upstream regulators modulate S6K kinase activity.
- Targeting S6K individually or in combination is a promising strategy for cancer therapies.

## Open Questions

- What are the functional differences between S6K isoforms in mediating tumorigenesis?
- What is the connection between the canonical roles of S6K and its noncanonical roles?
- How can S6K and its upstream kinases be targeted together to overcome negative feedback regulation?

## Introduction

The balance and tight control of cell size and cell numbers are two essential aspects for organ formation biologically and tumor growth pathologically. Of note, cell size has been found to be tightly controlled by mTORC1 by phosphorylating its diverse downstream substrates [1], in particular the S6K kinase, whereas cell numbers have been preliminarily dictated by the Hippo pathway by modulating the key regulator yes-associated protein 1 (YAP) [2]. Interestingly, the cross talk between these two pathways has been recently reported, highlighting the notion that disruption of their balance will result in tumorigenesis [3]. Due to the critical roles of the mTORC1 and Hippo pathways under pathobiological conditions, studies and reviews investigating mTORC1 and YAP have been well established [4, 5]. However, a summary of the upstream regulation and downstream functions of S6K has still not been provided.

As one of the most well-established mTORC1 downstream effectors, S6K is directly phosphorylated by mTORC1 upon diverse stimuli, such as amino acids [6] and insulin [7]. Many studies have indicated that amino acid availability and insulin stimulation are both required for mTORC1 activation [5]. On the one hand, insulin binds its ligand to activate the phosphoinositide-3-kinase (PI3K)-AKT pathway, in turn regulating the tuberous sclerosis complex 1/2 (TSC1/2)-Ras homolog enriched in brain (Rheb) axis to release mTORC1 repression [7]. On the other hand, upon amino acid stimulation, the activated Rag GTPase heterodimer, containing Rag A/B with Rag C/D, binds to the regulatory associated protein of mTOR complex 1 (Raptor) and recruits mTORC1 to the lysosomal membrane, where it colocalizes with Rheb, leading to subsequent activation [8]. Specifically, upon amino acid deficiency, GAP activity toward the rags 2 and 1 (GATOR2-GATOR1) complex was identified to be in response to the suppression of Rag GTPases and subsequent mTORC1 inactivation [6]. Subsequently, activated mTORC1 phosphorylates S6K and 4E-binding protein 1 (4E-BP1), leading to protein synthesis, cell cycle progression, and glucose homeostasis [9, 10]. Consistent with these findings, dysregulation of PI3K-AKT-mTOR-S6K components is predominantly linked to a large proportion of human malignancies, including breast, kidney, prostate, and liver cancers [11–13]. More interestingly, activation of the mTORC1-S6K pathway attenuates insulin-stimulated AKT activity in certain tumors through negative feedback phosphorylation of specific substrates [14].

Beyond controlling cell size, S6K has recently attracted more attention due to its important roles in cellular homeostasis [15, 16]. Thus, whether and how other stimuli, except via mTORC1, regulate S6K and how S6K plays distinct roles in addition to controlling cell size by targeting other downstream substrates are not well summarized. Hence, we comprehensively summarize the emerging upstream regulators, downstream substrates, mouse models, and clinical relevance for S6K and highlight employing S6K inhibitors for precision cancer therapies.

## S6K in tumorigenesis

**Fig. 1:**
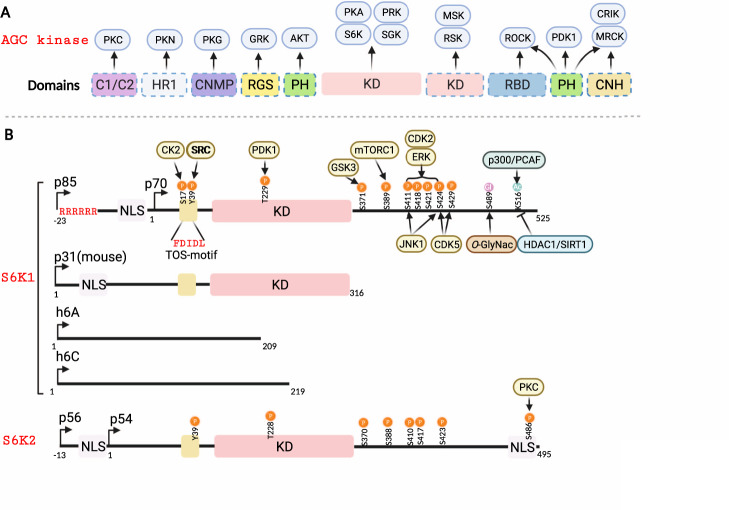
Protein structure and functional domains for S6Ks. **A** Conserved features of AGC family kinases. AGC family kinases are divided into 21 subfamilies due to the conserved functional regions beyond the kinase domain. **B** Alternative translation start sites of S6K result in several isoforms of S6K proteins. S6K1/2 share more than 80% similarity in their amino acid sequences, so they are often subject to similar posttranslational modifications (PTMs) but also with observed differences among isoforms. C1/C2, conserved domain 1 or 2; HR1, heptapetide repeat 1; CNMP, cyclic nucleotide monophosphate-binding; RGS, regulator of G protein signaling; PH, pleckstrin homology; KD, kinase domain; RBD, Rho-binding domain; CNH, citron homology; P, Gl, and Ac represent phosphorylation, glycosylation, and acetylation, respectively.

S6K belongs to the AGC kinase families termed cAMP-dependent protein kinase 1 (PKA), cGMP-dependent protein kinase (PKG), and protein kinase C (PKC) [17]. AGC kinases are serine/threonine protein kinases that evolutionarily share several conserved structural characteristics (Fig. 1A) and are involved in various important cellular processes [18]. AGC kinases are composed of 63 members, including AKT, phosphoinositide-dependent kinase 1 (PDK1), S6K, and large tumor suppressor kinase (LATS) [19]. Aberrations of AGC kinases, including mutations/amplifications of *AKT*, *PKC*, *S6K1*, and serum/glucocorticoid-regulated kinase 1 (*SGK1*), often occur under various pathological conditions, especially in cancer development and progression [20]. As a result, a broad range of AGC kinase inhibitors, such as agents targeting AKT, S6K, and PKC, have been adopted in clinical trials to explore their potential in cancer therapies [21].

### Members and functional domains of S6K

S6K contains two members, S6K1 and S6K2, encoded by *RPS6KB1* on chromosome 17 and *RPS6KB2* on chromosome 11 in humans, respectively [22]. Given the alternative translation start sites, transcripts of both genes include two isoforms: p70 S6K (S6KαII, 502 aa) and p85 S6K (S6KαI, 525 aa) for S6K1, as well as p54 S6K (S6KβII, 482 aa) and p56 S6K (S6KβI, 495 aa) for S6K2 (Fig. 1B). Of note, the longer forms of S6K obtain a putative nuclear localization signal (NLS) within additional amino acids in the N-terminus than the shorter forms. The p56 S6K2 isoform is actually located in the nucleus because of its N-terminal NLS, while the p54 isoform shuttles between the cytoplasm and nucleus in response to growth factor stimuli [23]. However, previous studies have shown that the p85 S6K1 isoform primarily resides in the cytoplasm and that the p70 isoform is located in both the cytoplasm and nucleus [24–27]. Interestingly, a recent finding indicated that via its N-terminal six-arginine motif, p85 S6K, but not p70 S6K, could be secreted from cancer cells into microenvironments and educate surrounding cells to confer malignant breast cancer behaviors [28]. In addition, alternative splicing of the *RPS6KB1* gene generates a shorter isoform of S6K, namely, p31 S6K1 in mice (and two isoforms in humans, namely, h6A and h6C), which resides in the nucleus and may function as oncoproteins [24, 29, 30].

S6K1 and S6K2 share more than 80% amino acid sequence identity, especially within their kinase domains. They also manifest a similar modular structure comprising an N-terminal regulatory region that contains a TOR signaling (TOS) motif, kinase domain (KD), and C-terminal regulatory region (containing a pseudosubstrate domain) (Fig. 1B). However, accumulating studies have revealed that S6K1 and S6K2 might play distinct roles in cells in part due to minor differences [22, 31, 32]. Specifically, the PDZ domain in S6K1 might associate with the cytoskeleton by binding to neurabin [32]; in contrast, an additional NLS and a proline-rich region [26] in S6K2 might facilitate diverse cellular localization and protein interactions [22]. Similar to other AGC kinases, such as AKT and SGK, S6K also recognizes and phosphorylates a conserved motif: (R)xR/KxxS/T containing Ser/Thr to be phosphorylated preceded by Arg/Lys at position -3 and alternatively another Arg/Lys at position -5. As such, S6K phosphorylates distinct substrates to regulate protein synthesis, transcription, cell proliferation, cell metabolism, and survival [31, 33], which will be further summarized in the following sections.

### Biological functions of S6K in metabolism, aging, and neuro-related diseases

In metabolic regulation, deletion of *S6k1/2* causes activation of AMPK in multiple tissues to control the energy state of the cell and the AMPK-dependent metabolic program [34]. Many studies have revealed that caloric restriction protects against age-related pathologies and increases lifespan in various animal models, such as yeast, *C. elegans*, and mice [35]. Consistently, depletion of *S6k1* resembles gene expression patterns of caloric restriction or prolongs lifespan by pharmacological activation of AMPK in mice [36], indicating a potential function of S6K in aging. Meanwhile, S6K1 also plays an important role in glucose metabolism via feedback regulation of insulin receptor substrate 1 (IRS1) to improve glucose tolerance and insulin sensitivity in liver-specific and systematic *S6k1*-deficient mice [37, 38]. Given the impacts of mTOR in neurodegenerative diseases, targeting S6K suggests a potent strategy for patients to achieve better outcomes [39].

### Clinical relevance of S6K in tumorigenesis

Regarding its essential function in cell growth, cell cycle, and cell metabolism, dysregulation of S6K occurs in various malignancies, including but not limited to breast [22], ovarian [40], hepatic [41], and prostate [42] cancers. Previous studies revealed that both *RPS6KB1* and *RPS6KB2* exhibit amplifications or gene gains in breast cancer tissues [31, 43]. However, S6K1 and S6K2 might display distinct functions in specific breast cancers [22]. In ovarian cancer, S6K1 persists in an active state, which is essential for the initiation and progression of tumors [44]. It has also been reported that dendriplex nanoparticle delivery of siRNA targeting S6K1 could potently diminish the stemness and metastatic properties of ovarian cancer stem cells [40]. Consistent with this finding, increased transcripts of *RPS6KB1* are considered an independent prognostic marker for poor prognosis of hepatocellular carcinoma patients [41]. S6K1 also participates in the development of brain tumor pathogenesis [45], and the gene expression patterns of S6Ks have been evaluated in brain and central neuron system tumors, indicating that S6K1 rather than S6K2 exhibits elevated expression in brain tumors and is associated with poor prognosis of patients [41]. Lu et al. reported that the phosphorylation level of S6K1 was closely related to TNM staging and lymph node metastasis of esophageal squamous cell carcinoma [46]. Through IHC staining analyses, Du et al. demonstrated that the expression of S6K1 and phosphorylation of S6 were markedly elevated in human vascular tumors, suggesting mTORC1 inhibitors as promising agents for cutaneous vascular lesions [47].

In contrast to *S6K1*, *S6K2* amplification is a common event in gastric cancer, indicating a poor outcome [48]. S6K2 has also been suggested as an effector under fibroblast growth factor-2 (FGF2) stimulation, inducing the survival of small cell lung cancer cells and chemoresistance via the formation of a complex with B-Raf and PKCε [49]. In hepatocellular carcinoma (HCC), S6K overexpression is positively correlated with tumor nuclear grade and inversely correlated with tumor size [50]. Moreover, S6Ks also play key roles in the progression, survival, migration, and response to chemotherapy drugs of prostate cancer, indicating that S6K is a promising target for advanced prostate cancer treatment [42].

### Genetic mouse models for S6K

Given that S6K functions as a pivotal regulator in the context of both physiological and pathological conditions, various transgenic or knockout mouse models have been developed to study corresponding diseases (Table 1). Pende et al. showed that mice depleted of *S6k1* or *S6k2* were viable, while double knockout of *S6k1/2* (*S6k1/2*^*−/−*^) resulted in a sharp reduction in survival because of perinatal lethality [51]. In addition, compared with WT mice, *S6k1*^*−/−*^ mice exhibited a markedly smaller size, while *S6k2*^*−/−*^ mice were slightly larger. Although S6K2 may play a dominant role in the process, S6K1 and S6K2 are both required for the maximum phosphorylation of S6. However, S6 phosphorylation at S235 and S236 remained in *S6k1/2*^*−/−*^ cells, which might be maintained by 90-kDa ribosomal protein S6 kinases (RSKs) [51–53].

**Table 1:** Genetic mouse models for S6K kinases.

Depletion of Gene(s)	Manifestations/diseases	References
*S6k1*^*−/−*^	Viable; smaller size; decreased S6 phosphorylation	[51]
*S6k1*^*−/−*^	Hypoinsulinemia, glucose intolerance, and less insulin secretion	[58]
*S6k1*^*−/−*^	Responsive to high-fat diet	[38]
*S6k1*^*−/−*^	Increased lifespan and reduced age-related pathologies	[36]
*S6k1*^*−/−*^	Retarded self-renewal of murine hematopoietic stem cells, and prolonged survival of mice	[56]
*S6k1*^*−/−*^	No difference in phenotypes of Huntington’s disease when crossed with R6/2 mouse model	[60]
*S6k1*^*−/−*^	Reduced incidence of renal hypertrophy and diabetic renal hypertrophy. Rapamycin had no impact on the renal hypertrophy induced by uninephrectomy of S6K1^−/−^ mice.	[62]
*S6k1*^*+/−*^	Reduced amyloid-β and tau pathology and improved synaptic plasticity and spatial memory in mice with Alzheimer’s disease	[61]
Liver *cS6k1*^*−/−*^	Improved insulin sensitivity and glucose tolerance after high-fat diet	[37]
*S6k2*^*−/−*^	Viable; slightly larger; dominant decrease in S6 phosphorylation	[51]
	Increased ketogenesis	[16]
*S6k1/2*^*−/−*^	Perinatal lethality, a sharp reduction of viability; responsive to mitogen and rapamycin	[51]
	No impact on the progression of cardiac hypertrophy induced by pathological, physiological factors, or transgenic activation of IGF1R-PI3K	[66]
Liver *cS6k1/2*^*−/−*^	Retarded ribosome biogenesis; resembled to S6^P−/−^ knock-in mice	[55]

Recently, ribosome biogenesis has emerged as a promising vulnerability for cancer treatment due to its essential role in cell transformation and tumorigenesis [54]. It has been reported that depletion of both *S6k1* and *S6k2* in the liver significantly retards the ribosome biogenesis transcriptional program, but has no influence on the recruitment of RNAs into polysomes [55]. Knockdown of *S6k1* impedes the self-renewal potential of murine hematopoietic stem cells, and depletion of *S6k1* shows prolonged survival in mice, suggesting a potential strategy for myeloid malignancy treatment [56]. Implementing xenograft models in *S6k1*^*−/−*^ and *S6k2*^*−/−*^ mice, Lee et al. observed that depletion of tumor stroma *S6k1*, but not *S6k2*, retarded tumor growth and angiogenesis, accompanied by impaired mRNA transcription and protein expression of HIF1α and its target genes [57]. This observation highlights S6K1 as a key regulator in the reconstruction of tumor microenvironments in favor of tumor growth.

In addition to cancer, *S6ks* knockout mice have also been employed to investigate several other pathologies. Interestingly, upon high-fat diet, *S6k1* KO mice exhibited increased glucose and free fatty acid levels coupled with markedly enhanced β-oxidation and metabolic rates but resist weight gain [38]. For the insulin response, high-fat diet-mediated excess nutrients might potentiate mTORC1-S6K activation, leading to IRS1 phosphorylation and degradation, thus desensitizing insulin signaling [38]. In contrast, *S6k1* KO mice exhibited lower IRS1 phosphorylation and a normal response to insulin signaling. Simultaneously, S6K-mediated S6 phosphorylation-deficient knock-in mice (*S6*^*P−/−*^) phenocopied *S6ks* KO mice in reduced ribosomal biogenesis transcriptional programs [55]. As reported, *S6k1*^*−/−*^ mice exhibited hypoinsulinemia and glucose intolerance accompanied by decreased insulin secretion, which is mildly phenocopied by *S6*^*P−/−*^ mice [58, 59]. It has been reported that the gene expression patterns of *S6k1* depletion mice resemble those of caloric restriction or pharmacological activation of AMPK to prolong the mouse lifespan [36]. In hepatic steatosis, after high-fat diet feeding, liver-specific *S6k1* knockout mice exhibited improved glucose tolerance and systemic insulin sensitivity, with consistent food intake and body weight compared with their wild-type (WT) counterparts [37]. In addition, *S6k2*^*−/−*^, but not *S6k1*^*−/−*^ mice, exhibited increased ketogenesis [16]. Mice generated by crossing *S6k1*^*−/−*^ mice with the R6/2 mouse model, an acknowledged model for Huntington’s disease, did not show any improvements in behavioral or physiological phenotypes [60]. Caccamo et al. reported that in a mouse model of Alzheimer’s disease (AD), depletion of one copy of *S6k1* significantly reduced amyloid-β and tau pathology, two neuropathological hallmarks for AD, accompanied by improvements in synaptic plasticity and spatial memory [61]. Moreover, rapamycin suppressed 60–70% hypertrophy in WT mice, but failed to rescue the 10% hypertrophy in *S6k1*^*−/−*^ mice, suggesting that S6K1 plays an essential role in the progression of compensatory renal hypertrophy and diabetic renal hypertrophy induced by uninephrectomy [62]. Ohanna et al. showed that depletion of *S6k1*, but not *S6k2*, evidently reduced the size of myoblasts, as well as rapamycin sensitivity, but left myoblast cell proliferation intact. In the differentiated state, *S6k1*^*−/−*^ myotubes also exhibited smaller sizes but normal nuclei, which abolished IGF1-induced hypertrophy [63]. Previous studies also showed that cardiac hypertrophy could be caused by pathological and active forms of PI3K, which might be related to S6K1 activation [64, 65]. However, deletion of both *S6k1* and *S6k2* demonstrated no obvious impact on the progression of cardiac hypertrophy [66], indicating the more complicated role of S6K in cardiac hypertrophy.

Except for these mouse models directly manipulating S6K (Table 1), several other genetic mouse models targeting *mTOR, Tsc1/2, Akt*, and *Pdk1* may heavily correlate with their regulatory functions on S6K directly or indirectly, which have been well summarized previously [67–70].

## Upstream regulators of S6K

### Genomic and transcriptional regulation of S6K

Genes encoding S6Ks located in human chromosome 17q23 displayed amplification in breast cancers along with other colocalized genes, including *PAT1*, *TBX2,* and *RAD51C*, jointly leading to a worse outcome [71]. Single nucleotide variants induced by missense mutations might influence protein functions. D14N and E44Q mutants of S6K1, with allele frequencies of 0.03282% and 0.0008244%, respectively, have been identified from the SPARK autism database and Simon Simplex Collection. The S6K1 D14N mutation results in enhanced de novo translation, spontaneous neural fate specification, and impaired dendritic arborization. Importantly, autistic individuals with the D14N mutation exhibit lower IQ scores than unaffected siblings [72]. Although the E44Q mutation shows increased translation in neurons, neuronal morphology is intact. This finding underscores the importance of translation control dictated by S6K1 in neural development and plasticity [72].

At the transcriptional level, Holz et al. revealed that estrogen receptor (ER) could bind to the proximal promoter of the *S6K1* gene assisted by GATA3 in ER-positive breast cancer, controlling the proliferation of breast cancer cells [73]. As a key regulator of cellular energy metabolism, estrogen-related receptor alpha (ERRα) also acts on the promoter of the *S6k1* gene, inhibiting its transcription to sensitize ERα-negative breast cancers to mTORC1/S6K1 inhibitors [74]. Moreover, serine and arginine-rich splicing factor 1 (SRSF1) alternatively spliced S6K1 and yielded shorter variants named p31 S6K1, which lacked most of its catalytic domain [29, 30]. It has been revealed that Sam68, an RNA-binding protein, can bind to mRNA encoded by *RPS6KB1* and suppress alternative splicing by SRSF1 [75]. Upon *Sam68* deficiency, SRSF1-mediated splicing resulted in the expression of p31 S6K1, which dampened lipogenesis in adipocytes [75].

**Fig. 2:**
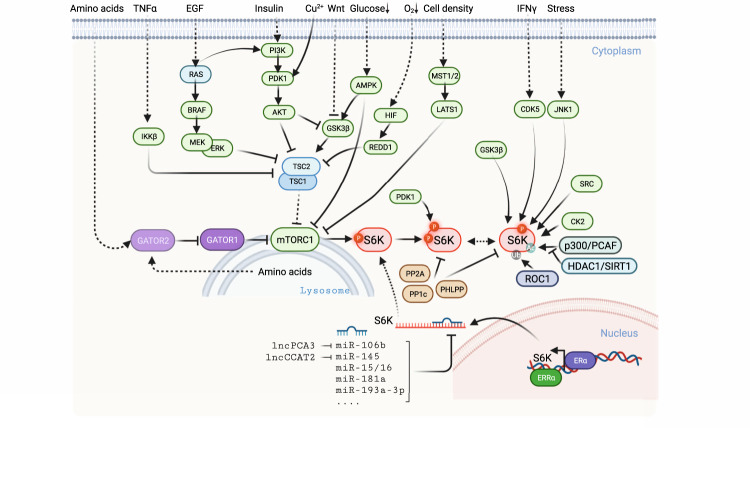
Upstream regulators of S6K. At the mRNA level, after transcription by ERα and ERRα, S6K mRNAs undergo miRNA-mediated degradation. At the protein level, several kinases are responsible for the release of the autoinhibitory domain of S6Ks, which enables accessibility for mTORC1 and PDK1. Upon various stimuli, including amino acids, cytokines, and metal ions, TSC1/2 lose the ability to suppress mTORC1 activity. Along with phosphorylation by mTORC1, PDK1 induces phosphorylation of S6Ks and results in its full activation. In contrast, phosphatases, including PP2A, account for the dephosphorylation of S6Ks. In addition, S6Ks are subject to ubiquitination and acetylation by ROC1 and P300/PCAF, respectively.

In terms of S6K mRNA, Ramaiah et al. reported that the miR-15/16 complex was responsible for the *RPS6KB1* degradation in MDA-MB-231 cells, regulating cell proliferation and survival [76]. In prostate cancers, upregulated Snail suppressed the transcription of miR-128, a negative regulator of S6K1 mRNA, resulting in increased expression of S6K1, hypoxia-inducible factor 1 subunit alpha (HIF1α), and pyruvate kinase M 2 (PKM2) [77]. In addition, miR-195 was proven to play a suppressive role in prostate cancer development by targeting S6K1. Re-expression and knockdown of S6K1 abolished and resembled the effects of miR-195, respectively, both of which were closely correlated with the progression and prognosis of prostate cancer patients [78]. Yao et al. demonstrated that miR-193a-3p was responsible for the quality control of S6K2 mRNA in non-small cell lung cancer (NSCLC), which might serve as a tumor suppressor and suppress the metastatic traits of NSCLC [79]. Furthermore, miR-557 and miR-3182 were identified as regulators of the degradation of S6K1 mRNAs in TNBC [80]. miR-506-3p could bind to the 3’-UTR of S6K1 mRNA, leading to its degradation and subsequently increasing insulin sensitivity [81]. Wang et al. reported that miR-181a downregulated S6K1, resulting in spermatogenesis, an uncommon function of S6K1 [82]. In addition to miRNAs, long noncoding RNAs (lncRNAs) have also been implicated in abrogating the regulation of S6K expression by miRNAs. Of note, lncRNA PCA3 disrupted the binding of miR-106b with its target genes, including S6K1, in ovarian cancer cells; [83] lncRNA CCAT2 was responsible for radiotherapy resistance by downregulating miR-145-S6K1 in esophageal cancer cells [84] (Fig. 2).

### Phosphorylation modification of S6K

The kinase activity and subcellular localization of S6K are predominantly regulated by multiple-site phosphorylation events in response to diverse extracellular stimuli, including growth factors, nutrients and mitogens, which cooperate with each other to fully activate S6Ks (Figs. 1B and 2). Over recent decades, a stepwise activation model of S6Ks has been widely accepted, including a conventional and an alternate model [33]. Unlike other AGC family kinases, S6Ks possess an autoinhibitory domain, which blocks the activation loop from mTORC1 and PDK1, resulting in an inactive form of S6K [33]. The release of the activation loop is achieved by phosphorylation at several sites (S411, S418, T421, and S424) on its C-terminal autoinhibitory domain by members of MAPK and CDK1 under mitogen stimuli [85]. It is worth noting that phosphorylation of S6K by JNK1 at S411 and S424 might account for its degradation only when IKKβ is inhibited [86].

It is widely known that upon the stimulation of insulin, activation of the PI3K-AKT pathway results in the activation of mTORC1, which is responsible for the phosphorylation of the hydrophobic motif of S6Ks (S6K1 at T389 and S6K2 at T388) [87]. In parallel, activation of PDK1 is responsible for a subsequent phosphorylation event on the activation loop of S6Ks (S6K1 at T229 and S6K2 at T228) [88], finally leading to full S6K activation. Conceivably, pathways and messengers that converge onto mTORC1 or PDK1 lead to the activation of S6Ks to control downstream cellular progression.

In the upstream regulation of mTORC1, the TSC complex, acting as a GTPase, plays the most important repression role as a central hub [89] (Fig. 2). Upon stimulation with growth factors, active AKT directly phosphorylates and inhibits TSC2 [90, 91]. Similarly, following growth factor-induced Ras activation, the MEK-ERK pathway is capable of phosphorylating and suppressing TSC2 [92, 93]. Under stimulation of TNFα, IKKβ directly phosphorylates and suppresses TSC1 in breast cancer [94]. However, hypoxia can enhance TSC GAP activity via the induction of regulated DNA damage and development 1 (REDD1) [95]. In contrast, energy deficiency-mediated activation of AMPK directly phosphorylates the mTORC1 subunit Raptor and indirectly phosphorylates and activates TSC2, jointly abrogating mTORC1 activity [91, 92]. LATS1/2, the key components of the Hippo pathway, can directly phosphorylate the mTORC1 core subunit Raptor to counteract the interaction with Rheb, resulting in the downregulation of mTORC1-S6K signaling [3]. Apart from directly phosphorylating S6K1 by glycogen synthase kinase 3 (GSK3) [96], S6K also regulates GSK3 by inactivating AKT kinase via a feedback loop [97]. Interestingly, Wnt and energy signals also modulate TSC2 via GSK3 and AMPK-mediated phosphorylation [98]. Except for the insulin-mediated PI3K-AKT pathway, amino acid availability also essentially contributes to mTORC1 activation via its sensors, the GATOR2-GATOR1 complex and Rag GTPases [6]. Thus, signals regulating these components will also modulate mTORC1 kinase activity and subsequently S6K phosphorylation [8, 99, 100]. Unlike mTORC1, the upstream regulation of PDK1 has been far behind due to the properties of autophosphorylation and activation [101–104]. In addition to PIP_3_ assisting PDK1 binding with its substrate AKT, copper has also been identified to assist PDK1 binding with its substrates, such as S6K and AKT, resulting in the activation of oncogenic pathways [105].

It is of great interest that with treatment with IFN-γ for myeloid cells and insulin for adipocytes, CDK5 could directly phosphorylate S6K1 on its C-terminal domain at S424 and S429 along with phosphorylation at T389 by mTORC1. Furthermore, CDK5-mediated phosphorylation causes a conformational change in S6K1 and leads to a higher affinity for targets related to lipid metabolism than its canonical target S6 [106]. In neurons, CDK5 can also phosphorylate S6K1 on the autoinhibitory domain at S411, which facilitates S6K1 activation to impact spine morphogenesis [107]. As we mentioned above, GSK3 can also directly phosphorylate S6K1 on its turn motif at S371, which is antagonized by phosphatase 2A (PP2A), resulting in the activation of S6K1 [96]. This finding provides evidence for the usage of GSK3 inhibitors for diabetes or cancers harboring dysregulated S6K1.

In addition to influencing kinase activity, phosphorylation could participate in other aspects of the regulation of S6Ks (Figs. 1B and 2). For example, PKC is able to phosphorylate S6K2, but not S6K1, at S486 located within its NLS in the C-terminus, thus abrogating the function of the NLS and leading to retention of active S6K2 in the cytoplasm [108]. Moreover, casein kinase 2 (CK2) phosphorylates S6K1 on its N-terminus at S17, which enhances S6K1 phosphorylation and thus leads to a cytoplasmic pool of S6K1 [109]. S6Ks also undergo tyrosine phosphorylation by platelet-derived growth factor receptor (PDGFR)/Src, yet its mechanism remains unknown [110].

Similar to the regulation of AKT, both phosphatase PP2A and PHLPP are involved in the dephosphorylation of S6K1 [111, 112]. Among them, the B regulatory subunit of PP2A (PP2A-B) in *Drosophila* and its homolog PPP2R5C in humans have been identified to interact with S6K and specifically target the PP2A holoenzyme to dephosphorylate S6K [113]. The vitamin D receptor, PP1c or PP2Ac, can assemble a ternary complex with S6K1 to dephosphorylate S6K1 [114]. PHLPP dephosphorylates S6K1 independent of the process of AKT dephosphorylation [115].

### Other regulatory modifications of S6K1

In addition to phosphorylation, S6Ks are also subject to ubiquitination and acetylation modifications (Fig. 1B). Notably, S6Ks, with ubiquitinated residues in the kinase domain, could be degraded through the 26S proteasomal machine [116]. Later studies revealed that ROC1 was the potential corresponding E3 ligase for S6K1 ubiquitination [117], whereas E3 ligase-mediated S6K2 ubiquitination is currently unknown. Similar to ubiquitination, acetylation also plays a role in the regulation of S6K stability. S6K1 undergoes acetylation at K516 by p300 and p300/CBP-associated factor (PCAF) to stabilize the S6K1 protein under mitogen stimulation [118, 119]. Meanwhile, both HDAC and Sirtuin inhibitors could markedly enhance the acetylation level and protein abundance of S6K [118]. Of note, during calorie restriction, SIRT1 deacetylated S6K1 in intestinal stem cells (ISCs), thereby enhancing its phosphorylation via mTORC1, leading to ISC self-renewal [120]. In addition, under high glucose, S6K acetylation was eliminated by HDAC1, contributing to kidney glomerular cell hypertrophy and matrix expansion [121]. In addition, S6K1 was also modified by *O*-GlyNAc transferase (OGT) at S489 to suppress mTORC1-S6K1 signaling, likely impairing the transcription of proinflammatory genes in macrophages [122].

## Downstream substrates and biological roles of S6K

**Fig. 3:**
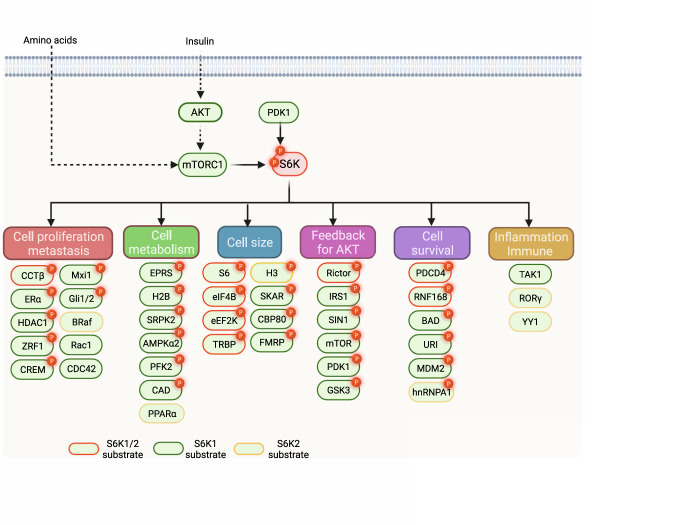
Downstream substrates and effectors of S6K. After phosphorylation and activation by mTORC1 and PDK1, S6Ks function in dictating various physiological and pathological processes, including cell size control, metabolic homeostasis, survival, and metastasis, via a variety of downstream substrates in a phosphorylation-dependent or phosphorylation-independent manner. Although S6Ks hold several mutual substrates (labeled red), an increasing amount of evidence suggests that distinct functions are exerted by S6K1 (labeled green) and S6K2 (labeled orange), in part due to the minor difference in amino acid sequences.

With the accumulation of downstream substrates, the functions of S6Ks have been expanded in many biological processes, including canonical cell size control, cell metabolism regulation, cell growth, and anti-apoptosis, as well as negative regulation of S6K upstream signals (Fig. 3).

### Contribution to cell size

**Fig. 4:**
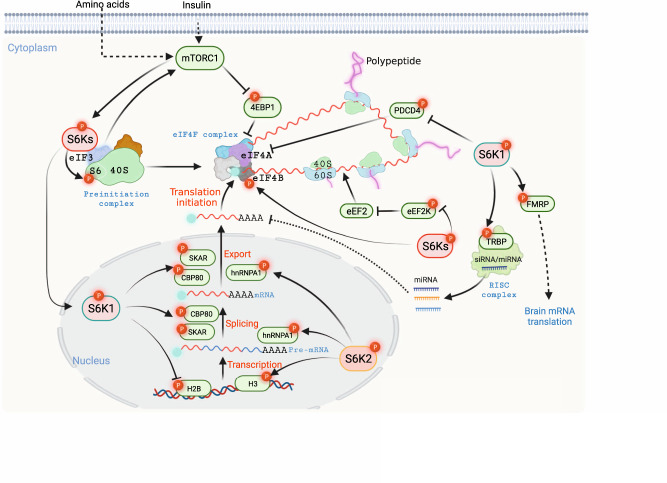
S6K controls cell size by directing multiple processes of protein synthesis. Phosphorylation of histones by S6K may dictate specific gene transcription, followed by phosphorylation and activation of CBC and EJC components, which play key roles in pre-mRNA maturity and exportation. S6Ks also function in preinitiation complex formation and translation initiation by phosphorylating eIF3, S6, eIF4B, and PDCD4, resulting in efficient initiation of protein synthesis. On the other hand, during elongation, phosphorylation of eEF2K by S6Ks favors an active state of eEF2, resulting in efficient ribosome translocation and protein translation.

As the most well-established substrate of S6K, phosphorylation and activation of S6 play an important role in promoting protein synthesis (Fig. 4). There are two subunits of ribosomes in higher eukaryotes, the 40S (small) and 60S (large) subunits, which consist of rRNA and various ribosomal proteins. Among them, 40S ribosomal protein S6 could be phosphorylated by S6Ks on its C-terminus at five conserved serine residues (S235, S236, S240, S244, and S247) [123]. Of note, S6K2, but not S6K1, has been reported to be a primary kinase for S6 phosphorylation [51]. *rpS6*^*P−/−*^ knock-in mice, substituting these phosphorylatable serine residues into alanine, displayed a smaller cell size of mouse embryo fibroblasts and pancreatic β cells [55]. This might be derived from the effects of S6 phosphorylation on ribosomal biogenesis [55]. *rpS6*^*P−/−*^ mice also exhibited glucose intolerance, reduced insulin secretion, and hypoinsulinemia [59]. It is worth noting that *rpS6*^*P−/−*^ mice presented diminished long-term potentiation and reduced translation of mitochondria-related mRNAs limited to the nucleus. In contrast, *rpS6*^*P−/−*^ mice exhibited unchanged brain size, weight, and morphology, which was consistent with the finding that S6 phosphorylation by S6Ks promotes global protein synthesis [124]. Despite extensive reports about S6 as an S6Ks substrate to regulate protein synthesis, the biological function of S6 phosphorylation still needs to be further characterized. For example, the phosphorylation level of S6 at S235 and S236 was detectable, although with decreased intensity in *S6Ks*-deficient cells, which may suggest that other kinases function in tuning S6 phosphorylation and function [51]. Indeed, RSKs and CK1 were observed to responsive for the phosphorylation of S235/236 and S247, respectively [53, 125]. Therefore, it is necessary to be cautious when decoding S6K-S6 signaling. Interestingly, eukaryotic initiation factor 3 (eIF3) may function as an adaptor protein, where it associates with inactive S6K1 and recruits activated mTORC1 upon growth factor stimulation, leading to activation of S6K1 and subsequent phosphorylation of eIF4B for translation initiation (Fig. 4) [126]. The eIF3 preinitiation complex may guarantee the coordination of dynamic sequences followed by various stimuli [127].

The process of protein synthesis involves the initiation, elongation, termination, and recycling of ribosomes [128]. Initiation of translation is the key process for protein synthesis, which is under strict control via several regulatory mechanisms. Eukaryotic translation initiation factor 4B (eIF4B) plays a key role not only in recruiting ribosomes to the translation initiation complex but also in promoting the RNA helicase activity of eukaryotic translation initiation factor 4A (eIF4A) [126, 129], which is essential for the sufficient unwinding of mRNA for initiation of codon scanning. eIF4B is phosphorylated by S6Ks at S422, mutating which with Alanine impairs translation efficiency, indicating the important function of eIF4B phosphorylation by S6Ks [130]. Programmed cell death 4 (PDCD4), a known tumor suppressor, can bind with eIF4A by blocking the binding between eIF4A and eIF44G or suppressing eIF4A helicase activity, resulting in impaired translation [131]. Upon the stimulation of mitogens, S6K1 directly phosphorylates PDCD4, leading to β-TRCP-mediated degradation, which accounts for efficient protein translation and further cell growth [132]. In addition to initiation, the elongation phase is achieved by a cluster of eukaryotic elongation factors (eEFs), which also undergo phosphorylation regulation. S6Ks have been reported to phosphorylate eEF2 kinase (eEF2K) at S366, leading to inhibited eEF2 kinase (eEF2K) activity toward eEF2, which accounts for eEF2 dephosphorylation and being more active (Fig. 4) [133]. This S6K-eEF2K-eEF2 axis may serve as an accelerator of protein synthesis and cell growth under S6K activation. Via a proteomic analysis, Pavan et al. showed that the S6K1 interactome was predominantly associated with the “stress response” and “cytoskeleton” [134]. Indeed, under mildly stressful conditions, phosphorylated S6K1 and S6K2 are responsible for the phosphorylation of translation initiation factor 2α (eIF2α) at S51, which favors the assembly of stress granules (SGs). Specifically, S6K1 is the predominant kinase for eIF2α phosphorylation and subsequent SG formation, while S6K2 is vital for SG persistence [135]. Upon recovery from stress, protein synthesis resumes, which might protect cells from stressful insults.

Under insulin stimulation, S6K1 Aly/REF-like target (SKAR), acting as an adaptor protein, could be phosphorylated by S6K1, but not S6K2, at S383 and S385, which functions in recruiting S6K1 to the de novo mRNAs, possibly promoting the translation of specific messengers [136]. Further studies reveal that SKAR is a component of the exon-junction complex (EJC), the phosphorylation of which results in increased efficiency in pre-mRNA splicing and exportation to the cytoplasm, leading to enhanced translational efficiency [137]. Cell division cycle 42 (CDC42) is a GTP-binding protein that coordinates various cellular events through fine-tuned GTP binding and hydrolysis, including transcriptional regulation and cell cycle progression. The function of CDC42 in regulating pre-mRNA splicing is achieved by the nuclear RNA cap-binding complex (CBC) [138]. It is believed that S6K1 directly binds to and phosphorylates the CBC subunit CBP80, leading to enhanced RNA cap-binding activity [139], which possibly stimulates efficient splicing of pre-mRNA and subsequent mRNA translation. Serine/arginine-rich (SR) proteins are a family of RNA-binding proteins that are responsible for RNA processing by directly binding to the exons of pre-mRNAs and subsequently recruiting small nuclear ribonucleoproteins to enhance alternative splicing [140]. SR proteins are phosphorylated by SR protein kinases (SRPKs), which dictate their conformation, localization, and/or interactions with other proteins. Notably, SRPK2 undergoes S6K1-mediated phosphorylation, priming subsequent phosphorylation by CK1, which promotes its nuclear localization and SR protein phosphorylation (Fig. 4) [141]. Consequently, phosphorylated SR protein binds to small nuclear ribonucleoprotein U1 to promote the splicing of lipogenic pre-mRNAs.

Fragile X syndrome (FXS), a primary risk factor for the development of autism and retardation, is a consequence generated by loss-of-function of the RNA-binding protein FMRP, which controls ~3% of mammalian brain mRNA translation [142]. S6K1 has been identified as the predominant kinase responsible for FMRP phosphorylation in the mouse hippocampus, which contributes to FMRP activation and provides insights for the occurrence and treatment of Fragile X syndrome [143, 144]. In contrast to S6K1, S6K2 also plays a vital role in the nucleus. Ismail et al. identified that S6K2 could bind to chromatin and nuclear matrix cellular fractions and directly phosphorylate H3 at T45, which plays essential roles in the response to mitogens and cell proliferation and/or differentiation of leukemic cells [145]. In addition to S6K2, AKT also directly phosphorylates H3 at T45 upon DNA damage, which facilitates transcriptional termination for DNA damage-inducible genes, guaranteeing maximum transcriptional efficiency to maintain genomic integrity [146]. After transcription, precursor miRNAs need to be processed by the miRNA biogenesis machinery to assemble the RNA-induced silencing complex, which plays pivotal roles in mammalian development. Transactivating response RNA-binding protein 2 (TRBP), along with DROSHA, DGCR8, AGO2, and PACT, comprises the machinery for miRNA processing, which are often targets for phosphorylation regulation [147–149]. S6Ks have been reported to dictate the phosphorylation of TRBP, resulting in its optimal expression and promoting miRNA biogenesis in primary human dermal lymphatic endothelial cells (Fig. 4) [150].

### Contribution to feedback regulation of the PI3K-AKT pathway

**Fig. 5:**
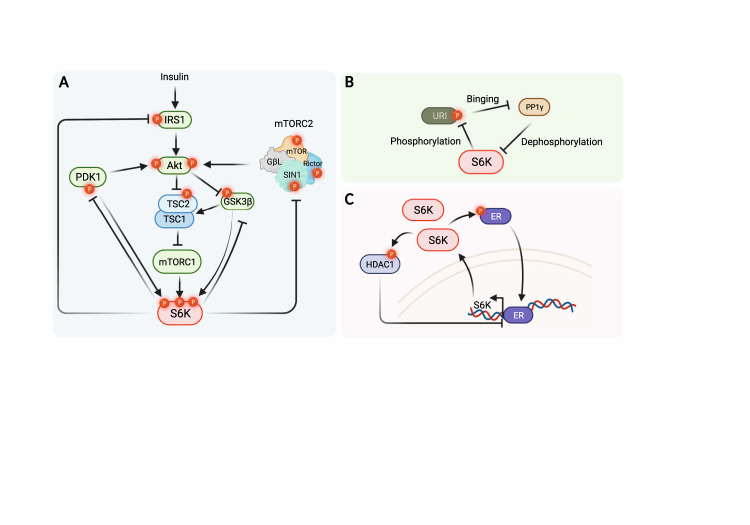
S6K functions in feedback regulation. **A** Feedback regulation of the PI3K-AKT signaling pathway. S6K could phosphorylate multiple sites of IRS1, leading to its degradation and insensitivity to insulin treatment. S6Ks also tune the activity, stability, or complex formation of PDK1 and mTORC2, key regulators of full AKT activation. Inhibition of AKT in turn will fail to release mTORC1 activity, resulting in subsequent suppression of S6Ks. **B** S6K1 is able to phosphorylate URI, leading to disassociation of the URI/PP1γ complex, which liberates the phosphatase activity of PP1γ that functions to inactivate S6K1. **C** ERα could bind to the promoter of S6K and promote its transcription; on the other hand, ERα is subject to phosphorylation by S6K1, which promotes the transcriptional activity of ERα. Phosphorylation and activation of HDAC1 achieved by S6K1 is responsible for hypoacetylation of histones, which results in compact chromatin and suppression of transcription of ERα.

Interestingly, as an mTORC1 inhibitor, rapamycin markedly enhances PI3K/AKT activity in various cell lines, which suggests a potential negative feedback of mTORC1-S6Ks [151]. With this observation, the feedback regulation of S6K to AKT has attracted extensive attention, which may account for a better exploration of the underlying mechanism of rapamycin-induced drug resistance in cancer therapies (Fig. 5A).

Upon insulin stimulation, IRS, an adaptor protein, could be recruited to the insulin receptor via its PH domain, capturing proteins containing the Src-homology (SH) domain, such as the p85 regulatory subunit of PI3K, followed by conversion of colocalized phosphatidylinositol (4,5)-bisphosphate (PIP_2_) to PIP_3_, finally leading to activation of the PI3K-AKT-mTORC1 pathway [152]. The initial observed pathway is S6K1-mediated phosphorylation of IRS1. Interestingly, *S6k1*-deficient mice sustain a regular level of glucose after fasting, suggesting hypersensitivity to insulin [58]. It has been reported that S6K1 can directly phosphorylate IRS1 at S307 and S636/S639, which abolishes the adaptor function of IRS1, leading to its insensitivity to upstream stimuli [38]. S6K-dependent inactivation of IRS also stems from the suppression of gene expression [153]. Subsequent studies revealed that residues S270, S307, S636, and S1101 within IRS1 are subject to phosphorylation by S6K1 directly under TNFα treatment, among which S270 might be the most important site [154, 155]. Under TNFα treatment, this phosphorylation event shows an increase in adipocytes of obese mice, possibly due to the inhibition of IRS activity and thus suppression of insulin-stimulated glucose uptake.

Upon exposure to upstream stimuli, mTOR can be phosphorylated at S2448 by active AKT, which then initiates a series of cellular responses by phosphorylating various mTORC1 substrates. Rapamycin treatment blocks serum-stimulated mTOR phosphorylation, which cannot be explained by repressing AKT. Additionally, after treatment with rapamycin, rapamycin-resistant S6K1 is capable of restoring S2448 phosphorylation and phosphorylating mTOR at S2446 within the reported regulatory repressor domain of mTOR [156, 157]. It is tempting to speculate that mTORC1 undergoes feedback regulation of S6K, but its function needs further investigation. As a key regulator of growth and proliferation in all eukaryotic cells, mTOR, along with LST8, Rictor, and stress‑activated protein kinase‑interacting protein 1 (Sin1), forms mTOR complex 2 (mTORC2), which perceives various stimuli and results in the phosphorylation of some AGC kinases, including the well-known phosphorylation and activation of AKT at S473 [129].

Interestingly, based on cellular context, both S6K1 and AKT1 could phosphorylate Sin1, causing the disassociation of Sin1 from other partners of mTORC2, which abolishes AKT activation [158]. This finding reveals a negative feedback to the activation of the PI3K/AKT pathway, provides a possible explanation for hyperactive AKT induced by aberrations of the mTORC1-S6K-Sin1 axis, and suggests corresponding strategies in tissue and cellular contexts. RPTOR independent companion of mTOR complex 2 (Rictor), another unique component of mTORC2, plays an important role in mTORC2-regulated cell growth, metabolism, and survival [92]. Rictor has also been observed to function as an E3 ligase coupled with Cullin1 and Rbx1, accounting for SGK1 ubiquitination and destruction [159]. Furthermore, a group of AGC family kinases, including AKT, SGK1, and S6K, has been validated to phosphorylate Rictor at T1135, which disassociates the Cullin1-Rictor complex but with minimal effect on the kinase activity of mTORC2 [159, 160]. Nevertheless, Rictor T1135A mutant cells show enhanced AKT phosphorylation dependent on mTORC2, suggesting that S6K1-dependent Rictor phosphorylation may impair mTORC2 activity [161]. Thus, this finding reveals another negative regulatory loop for mTORC1-S6K1-mTORC2 signaling.

PDK1 is another AGC family kinase and is known as an upstream kinase responsible for full activation of S6K together with mTORC1. Notably, our recent study revealed PDK1 as a bona fide substrate of S6K1 in a phosphorylation-dependent manner [162]. Briefly, S6K1 directly phosphorylates PDK1 on its PH domain at S549, which impedes its membrane localization by binding with adaptor protein 14-3-3 and then abrogates the interaction with and activation of AKT. Conceivably, mutations that abolish S6K1-mediated phosphorylation or 14-3-3 interaction with PDK1 are sufficient to promote AKT hyperactivation and facilitate tumorigenesis [162]. As discussed above, the TSC complex acts as a suppressor of mTORC1 and leads to the inhibition of S6Ks. As a direct substrate of AKT, GSK3 has been detected in persistent phosphorylation in tumor tissues and cells deficient in *TSC1/2*. Furthermore, phosphorylation of GSK3 is sensitive to rapamycin or deprivation of amino acids [97]. Zhang et al. revealed that S6K1 is responsible for GSK3 phosphorylation and subsequent inhibition in settings of growth factor deficiency. This feedback regulation on S6K for GSK3 enriches the complexity of the regulatory network for PI3K-AKT-mTORC1 signaling, which provides another explanation for a diverse range of diseases.

### Control of cell apoptosis

S6K1 also plays an essential role in the regulation of cell growth and survival, partially through the inhibition of cellular apoptosis. BCL-2 family proteins are responsible for the intricate regulation of apoptosis and are frequently subjected to PTMs to dictate cell death. BAD, a pro-apoptotic protein, has been reported to be phosphorylated by S6K1, which inactivated its pro-apoptotic function [163]. Indeed, rapamycin treatment could abolish IGF1-induced BAD phosphorylation and cell survival in *S6k*-deficient cells [163]. Unconventional prefoldin RPB5 interactor (URI) is a chaperone belonging to the prefoldin family that can associate with PP1γ in mitochondria and inhibit its phosphatase activity. Growth factors can disassociate URI/PP1γ and release the dephosphorylation activity of PP1γ, which can be blocked by rapamycin [164]. Indeed, S6K1 is a kinase responsible for URI phosphorylation at S372, after which discharged PP1γ is capable of dephosphorylating S6K1 at T389 [164], consisting of a negative feedback loop for delicate apoptosis control by sensing the status of homeostatic and stimulated conditions (Fig. 5B).

The cellular genome is continuously attacked by various factors, including irradiation and reactive oxygen species (ROS), after which cells employ a network of pathways to maintain genome integrity. p53, a gatekeeper for the genome, has been a “star molecule” for research in cell cycle arrest and apoptosis [165]. Mouse double minute protein 2 (MDM2) has been reported to be a primary E3 ligase for p53 degradation [166, 167]. S6K1 has been reported as a multifaceted modulator of MDM2. On the one hand, S6K1 phosphorylates MDM2, promoting its nuclear-cytoplasmic shuttling; on the other hand, S6K1 closely binds to MDM2, hindering ubiquitination of p53, which leads to p53 stabilization and transcription [168]. This finding bridges the metabolic/energy cues in the cellular response to DNA damage. In the process of DNA repair, the E3 ubiquitin ligase RNF168 can be recruited to DNA damage sites to generate and multiply histone ubiquitination signals, promoting the repair of DNA double-strand breaks. Apart from the regulation of p53 by phosphorylating MDM2, S6Ks directly phosphorylate RNF168, leading to its degradation mediated by TRIP12 and loss of its function in response to DNA damage signals, which finally results in genome instability [169].

In parallel, PDCD4 binds to the mRNA of X chromosome-linked inhibitor of apoptosis (XIAP) and Bcl-xL, suppressing the translation of XIAP and Bcl-xL. In response to FGF2 stimulation, S6K2 directly phosphorylates PDCD4, accounting for its degradation, which releases the translational inhibition of XIAP and Bcl-xL, favoring cell survival by apoptosis inhibition [170]. Heterogeneous nuclear ribonucleoproteins (hnRNPs), comprising at least 20 members, namely, hnRNPA1 to hnRNPAU, are essential for the alternative splicing, metabolism, and transportation of precursor mRNA [171]. FGF2-induced survival is also dependent on S6K2-mediated phosphorylation of hnRNPA1. Upon S6K2-dependent phosphorylation coordinated with other modifications, hnRNPA1 binds to BcL-xL and XIAP mRNAs, leading to their expression and prefering cell survival [172]. Interestingly, S6K2-dependent phosphorylation of hnRNPA1 is essential for the preferred splicing of the PKM2 isoform, favoring enhanced glucose expenditure and lactate production [173]. FGF2 has also been implicated in the antiapoptotic traits and chemoresistance of small cell lung cancer. It has been reported that S6K2, but not S6K1, forms a multiple protein complex with B-Raf and PKCε and functions in promoting the expression of antiapoptotic proteins, including XIAP and Bcl-xL, resulting in chemoresistance [49].

### Control of cell metabolism

AMP-activated protein kinase (AMPK) is a heterotrimeric complex composed of one catalytic subunit and two regulatory subunits that function in response to energy status. In the hypothalamus, suppression of AMPKα2 results in leptin-induced reductions in food intake and body weight [174]. Dagon et al. reported that S6K1 could heterodimerize with AMPKα2, phosphorylating it at S491, which increases food intake and weight gain [15]. AMPK, being a substrate of mTORC1-S6K signaling, adds more complexity to the regulation of metabolism and cell growth. Glutamyl-prolyl-tRNA synthetase (EPRS) has been reported to play an important role in regulating the protein interaction of tRNA synthetases and suppressing the translation of inflammatory protein mRNA [175, 176]. It has been found that EPRS is also subject to phosphorylation by S6K1 at S999, which promotes its release from the multi-tRNA synthetase complex and binding to fatty acid transport protein 1, assisting its membrane localization and leading to the uptake of long-chain fatty acids. This effect is evidenced by reduced weight and adipose tissue mass in homozygous *EPRS S999A* knock-in mice, which also exhibit a longer lifespan resembling that of *S6k1*-deficient mice [36].

6-phosphofructo-2-kinase (PFK-2) is responsible for the synthesis of fructose 2,6-bisphosphate, which functions in controlling glycolysis in various organs [177]. Upon insulin stimulation, PFK-2 goes through phosphorylation events at S466 and S483 by several kinases, including AKT and S6K1, which account for PFK-2 activation [178]. This phosphorylation event may result in increased kinase activity of heart isoenzyme (H-PFK2). Under insulin stimulation, phosphorylated H-PFK2 may directly enhance glucose transportation, promoting glycolysis in the heart [179]. During fasting, adipose tissues release nutrients, such as glycerol, into the circulation to sustain energy homeostasis, which is a process accomplished by the peroxisome proliferator-activated receptor (PPAR) family of transcription factors [180]. It has been proposed that instead of S6K1, S6K2 binds to nuclear receptor corepressor 1 (NCoR1), a corepressor of PPARα, leading to intact transcriptional activity of PPARα, which sustains hepatic energy homeostasis [16].

Upon adipogenic stimuli, S6K1 translocates to the nucleus and leads to the phosphorylation of histone H2B at S36, which accounts for the recruitment of the enhancer of zeste homolog 2 (EZH2) and subsequent trimethylation of H3K27. This epigenetic mark retards the transcription of the *Wnt* gene, leading to enhanced adipogenesis. Of note, obese humans and high-fat diet-fed mice exhibit detectable pS36 H2B and H3K27me3. In contrast, adipose tissue of mice depleted with *S6k1* shows lower levels of pS36 H2B and H3K27me3 [181], highlighting strategies to employ specific S6K1 inhibitors to suppress obesity and overcome insulin resistance, bypassing mTORC2 inhibition. In islet cells, S6K1 is capable of promoting H3K4me3 and inhibiting H3K27me3 to support transcription marker genes of β cells and suppress those of α cells [182]. It is believed that the pancreatic cell transition induced by S6K1 might provide basic clues for type I diabetes.

mTORC1-S6K1-SRPK2 signaling results in phosphorylation and activation of SR protein, leading to enhanced splicing of lipogenic pre-mRNAs, which suggests SRPK2 as a target for metabolic disorders induced by mTORC1. Under growth factor stimulation, the PI3K-AKT-mTORC1 signaling pathway is mobilized to dictate cellular anabolic processes and cell growth. It is well established that mTORC1 regulates mRNA translation, ribosomal biogenesis, and de novo lipid and sterol synthesis [55, 141, 183]. Through unbiased metabolomic profiling, Issam et al. revealed that mTORC1 plays an essential role in de novo pyrimidine synthesis via S6K1-mediated phosphorylation of carbamoyl-phosphate synthetase 2 (CAD) at S1859 [184], which is vital for de novo pyrimidine synthesis. Apart from lipid and protein synthesis, cells also adapt pyrimidine synthesis via the mTORC1-S6K pathway to respond to growth factor signals.

### Control of cell proliferation

In addition to charging cell size, metabolic homeostasis, and apoptosis to contribute to cell malignancies, S6K also tethers other signaling pathways to control cell proliferation and migration. For instance, S6K1 directly phosphorylates glioma-associated oncogenes (Gli1), a key effector of the Hedgehog signaling pathway [185], and unleashes Gli1 from its endogenous inhibitor SuFu to enhance its transcriptional activity and tumorigenesis capability [186]. Similarly, Gli2 also goes through S6K1-mediated phosphorylation and disassociation from SuFu, contributing to chondrocyte proliferation and differentiation [187]. These findings provide a rationale for the combined inhibition of the Hedgehog pathway and mTORC1-S6K axis in context-dependent diseases. On the other hand, S6K1 can also directly phosphorylate estrogen receptor alpha (ERα) and promote ERα-related transcription [188–190]. As mentioned above, ERα can transcriptionally promote S6K1 expression [73]. Thus, S6K1-ERα consists of a positive feed-forward relationship in dictating breast cancer cell proliferation, which may be adopted as a therapeutic target for breast cancer with *S6K1* amplification (Fig. 5C). Furthermore, histone deacetylase 1 (HDAC1), an essential epigenetic regulator that modulates chromatin structures, has also been identified as a substrate of S6K1 upon mitogen stimuli in breast cancer cells, which accounts for HDAC1 hyperphosphorylation and activation, leading to deacetylase-dependent suppression of ERα transcription. As such, the combination of mTOR and HDAC1 inhibitors exhibits a synergistic effect on the inhibition of breast cancer proliferation [191]. Thus, HDAC1 may function as a node for the S6K1-mediated negative feedback loop in the regulation of cell proliferation (Fig. 5C). Moreover, S6K1 has also been reported to directly phosphorylate MAX interactor 1 (Mxi1), a member of the MAD family capable of competing with MAX binding to c-Myc to suppress c-Myc-induced proliferation [192], and recruit the E3 ligase β-TRCP to degrade Mxi1 to unleash c-Myc oncogenic functions [193].

### Control of inflammation and immune response

Transforming growth factor β-activated kinase 1 (TAK1) is essential for the production of inflammatory mediators and plays a key regulatory role in innate immunity signal transduction mediated by cytokines and Toll-like receptors (TLRs). S6K1 has been reported to negatively regulate the TLR signaling pathway by inhibiting TAK1 kinase activity [194]. In detail, S6K1 competes with TAB1, an essential regulator of TAK1 catalytic activity, for binding to TAK1, which dampens TAK1-induced transcription of AP-1 and NF-κB target genes. Moreover, TAK1 could also inhibit S6K1 phosphorylation by disrupting its interaction with the mTORC1 subunit Raptor, thus inducing autophagy [195]. CD4^+^ T helper (Th) cells are essential for adaptive immune responses by secreting distinct cytokines. Among them, Th17 cells play a vital role in the development of chronic inflammation underlying rheumatoid arthritis, multiple sclerosis, and inflammatory bowel disease [196]. RORγ and Gfi1 are key regulators of the differentiation of Th17 cells, which account for the promotion and suppression of specific transcription events, respectively [197]. Kurebayashi et al. reported that S6K2, but not S6K1, is capable of binding with RORγ, promoting its nuclear localization and resulting in Th17 differentiation [198].

### Other functions

In addition to the above well-established functions, S6K also phosphorylates other substrates and performs certain functions. Upon activation of the adenylate cyclase signaling pathway, cAMP responsive element modulator (CREM) [199] undergoes S6K1-mediated phosphorylation [200]. Chaperonin containing TCP-1 (CCT) is also considered an S6K substrate and functions in protein folding. Mitogen-activated RSK and insulin-activated S6Ks all can converge on CCTβ by phosphorylating it at S260 in various mammalian cells [201]. Although deficiency of *S6ks* results in a smaller size and prolonged lifespan, the impacts of S6Ks on cellular senescence remain an unsolved problem. Recently, through a chemical genetic screen, Barilari et al. proposed that Zuotin-related factor 1 (ZRF1) is a direct substrate of S6K1. Knocking down or expressing nonphosphorylatable ZRF1 markedly impairs the inhibitory effects of S6K1 in senescence, which is evidenced by a sharp alteration of p16 levels, a cell cycle inhibitor and primary modulator of senescence [202]. Moreover, S6K has certain functions that are independent of its kinase activity. Notably, S6K1 directly binds to actin and promotes the activation of Rac1 and CDC42, leading to subsequent actin cytoskeleton reorganization and cell migration independent of its kinase activity [203]. Moreover, upon exposure to mitogen stimuli, S6K2, but not S6K1, binds with Yin Yang 1 (YY1) via its C-terminal region, but the physiological significance is still undefined [204].

## Inhibitors targeting S6Ks for cancer therapies

Given the multifactorial functions of S6Ks in controlling cell size, cell growth, cell apoptosis, and metabolic homeostasis, inhibitors targeting S6Ks and their upstream activators, such as mTORC1 and PDK1, hold promise for cancer therapies (Table 2).

**Table 2:** Inhibitors targeting S6Ks and related signals.

Target(s)	Inhibitor	Inhibition mode(s)	Features	References
S6K1	PF-4708671	Piperazinyl-pyrimidine compound	Decreased phosphorylation of S6	[205]
	PF-4708671		Sensitized resistant colorectal cancer cells to treatment of selumetinib	[206]
	PF-4708671		Suppressed cell invasion and proliferation in human lung cancer cell lines and tumorigenesis in nude mice	[207]
	PF-4708671		Improved corticospinal tract regeneration and locomotor recovery in central nervous system injury mice	[208]
	PF-4708671		Improved glucose homeostasis of high-fat diet-induced obese mice	[209]
S6K2	Compound 2	PF-4708671 derivative	Showed a high specificity to S6K2 over other kinases	[210]
S6K1	A77 1726	Active metabolite of Leflunomide	Released feedback suppression of IRS1, promoting the cell cycle arrest	[211]
S6K1	FS-115	Synthesized compound	Inhibited colony formation and sphere formation of breast cancer cells, suppressing primary tumor growth and distant metastasis of breast cancer in nude mice	[212]
S6K1	FL772	ATP competitive organometallic inhibitor	Reduced kinase activity of S6K1 but not of S6K2	[213]
S6K1	LY2584702	ATP competitive inhibitor	Phase I/Ib trials showed a decrease of total cholesterol and triglyceride levels, also a reduction in LDL and HDL cholesterol in serum	[214, 215]
	LY2584702		Multiple-dose study showed no intolerability, a dose-dependent reduction in LDL cholesterol, triglycerides, and Factor V activity in hypercholesterolemia volunteers	[216]
S6K1 and AKT	LY2780301	ATP competitive inhibitor	Phase I/Ib trials showed a linear PK with minor variability and irrelevant with problems of significant safety and tolerability when orally administered with 200 mg	[217, 218]
	LY2780301 and weekly Paclitaxel		Phase IB/II study showed tolerable side effects and with preliminary evidence of efficacy in patients with HER2-negative advanced breast cancers	[219]
S6K1	LYS6K2	Synthesized compound	Reduced cellular triacylglyceride level and apolipoprotein-B100 secretion in TSC-deficient cells	[220]
S6K1	AST	Natural product	Inhibited phosphorylation of S6 and IRS1, enhancing phosphorylation of AKT and S6K T389	[221]
Ret, Src, Raf, TOR, S6K kinases	AD80 and AD81	Synthesized compound	As polypharmacological agents, functioning in achieving an optimal balance by targeting various kinases	[222]
S6K1, AKT1/3	M2698	ATP competitive inhibitor	Dose-dependently impaired tumor growth and prolonged survival of mice with U251 glioblastoma	[223, 224]
	M2698 and trastuzumab or tamoxifen		Phase 1 study: M2698 was well tolerated; exhibited antitumor activity in advanced cancer patients who were resistant to multiple standard therapies	[225]
S6K1, JAK2	Gingerenone A	Natural compound	Suppressed cell growth and tumor growth in mice	[226]
	Gingerenone A		Enhanced insulin sensitivity and glucose uptake	[227]
mTOR	Temsirolimus, Ridaforolimus Everolimus	Rapalogs	Approved or underwent several clinical trials.Showed preferable water solubility and pharmacokinetic properties; suppressing the activation of S6K1 and 4E-BP1 via mTORC1 inhibition, subsequently resulting in cell cycle arrest and cell death	[228]
mTORC1/2	Streptomyces sp OA293	Ethyl acetate extracts	Showed potent inhibition of mTORC1 and AKT, suppressing activation of S6K1 and 4E-BP1	[229]
mTORC1/2	CC-223	ATP competitive inhibitor	Phase I dose-escalation study exhibited tolerable and manageable toxicities when patients were treated who had advanced cancer	[231]
	CC-223		Phase II study revealed highly durable tumor regression, marked reduction in NET carcinoid symptoms among well-differentiated neuroendocrine tumor (NET) patients	[232]
PI3K, AKT, mTOR	AZD8055 GDC0941 Selumetinib		Reduced cell proliferation and retarded tumor growth in PDX mice models of ovarian clear cell carcinoma	[233]
PDK1	SBF1	Allosteric inhibitor of PIF pocket	Predicted IC50 ranging from 2.0 to 10.0 μM	[234]
PDK1	OSU-03012	ATP competitive inhibitor; Celecoxib derivative	With IC50 variation in the low μM range	[235]
	OSU-03012		Showed induction of autophagy and ROS levels and subsequent suppression of cell growth in various cancers	[236]
PDK1	GSK470	Inhibition of T loop phosphorylation	Suppressed cell growth and enhanced cell death of multiple myeloma cells, which could synergize with proteasome inhibitor MG132	[237]
PDK1	JX06		Inhibited glycolysis and promoted cell apoptosis of multiple myeloma cells; synergized with proteasome inhibitor bortezomib	[238]
PDK1	BX795 and BX912	Inhibition of T loop phosphorylation	Abolished PDK1 dependent on Myc induction, resulting in cell death and a reduction in tumor sphere formation of various breast cancer cell lines	[239]
	BX795	Inhibition of T loop phosphorylation	Synergized with cisplatin to markedly reduce the epithelial-mesenchymal transition of oral squamous cell carcinoma	[240]
PDK1	Dichloroacetate		Synergized with NOS inhibitor T1023, leading to an evident reduction of tumor growth	[241]
AKT, PDK1	PHT-427	Binding with PH domains of AKT and PDK1	Enhanced oxidative stress levels and apoptosis in various cancers	[242, 243]

### S6Ks inhibitors

PF-4708671 is a cell-permeable piperazinyl-pyrimidine compound that specifically inhibits p70-S6K. Its inhibitory effects are evidenced by a prominent decrease in the phosphorylation of S6 after IGF1 stimulation, which also shows excellent selectivity for mitogen- and stress-activated kinases and RSK [205]. The effectiveness of PF-4708671 has also been validated in various settings. For example, S6K1 inhibition induced by PF-4708671 sensitizes colorectal cancer cells to selumetinib (an MEK1/2 inhibitor) treatment [206] to suppress cell invasion and proliferation [207] and improves corticospinal tract regeneration and locomotor recovery in central nervous system injury mice [208] and glucose homeostasis in high-fat diet-induced obese mice [209]. By targeting cysteine 150 of S6K2, but not S6K1, Gerstenecker et al. indicated that Compound 2 generated from PF-4708671 via a nucleophilic aromatic substitution reaction shows a higher specificity for S6K2 than other kinases, which may serve as a model for the development of next-generation S6K2-specific inhibitors [210]. Although the broad preclinical application of PF-4708671 as an S6K1-specific inhibitor has achieved promising results under diverse pathological conditions, no clinical trial regarding PF-4708671 has been conducted. Alternatively, A771726, an active metabolite of leflunomide approved by the FDA for curing rheumatoid arthritis, could inhibit S6K1 activity, release feedback suppression of IRS1, and promote cell cycle arrest in the S phase [211]. Furthermore, FS-115 potently suppressed the activity of S6K1, exhibiting marked selectivity for kinases from the AGC family. Treatment with FS-115 could also inhibit colony formation and sphere formation of breast cancer cells, suppress primary tumor growth, and reduce distant metastasis of breast cancer in nude mice [212].

ATP competitive inhibitors might be a powerful strategy for developing potent and specific suppressors for kinases. As an ATP competitive organometallic inhibitor, FL772 markedly reduces the kinase activity of S6K1 in the nanomolar range and exhibits more than 100-fold selectivity over S6K2 [213]. LY2584702 is another selective ATP competitive inhibitor for S6K1 against 83 other kinases. Phase I/Ib trials of LY2584702 indicate that the maximum tolerated doses are 75 mg twice daily or 100 mg once daily [214, 215]. Moreover, LY2584702 has been adopted in the treatment of dyslipidemia [216]. These phenomena may derive from the inhibition of S6K1 in the regulation of lipid metabolism. A single-ascending-dose study was performed to evaluate the safety, tolerability and pharmacokinetic (PK) features of LY2780301, an ATP competitive dual inhibitor of S6K1 and AKT, followed by a single oral dose. LY2780301 also shows a linear PK with minor variability and is irrelevant to problems of significant safety and tolerability when orally administered at 200 mg [217, 218]. Most recently, a TAKTIC study revealed that combined treatment with LY2780301 and paclitaxel (weekly) among patients with hormone-resistant HER2-negative advanced breast cancers showed a 6-month objective response rate (ORR) of 63.9% and 55% in all patients and patients with an activated PI3K/AKT pathway, respectively [219].

It has been reported that LYS6K2 could function as a selective inhibitor of S6Ks, resulting in the reduction of cellular triacylglyceride levels and apolipoprotein-B100 secretion in *TSC*-deficient cells [220]. As a xanthophyll carotenoid, astaxanthin (AST) is a natural product mainly derived from seafoods, functioning as an inhibitor of S6K1, which is evidenced by inhibited phosphorylation of S6 and IRS1. However, administration of AST is also accompanied by enhanced phosphorylation of AKT and S6K at T389 because of abolished negative feedback regulation of S6K1 on the PI3K/AKT pathway [221]. Combining genetics with chemistry, Dar et al. synthesized a series of compounds, among which AD80 and AD81 were identified as polypharmacological agents, possessing optimal balance against Ret, Src, Raf, TOR, and S6K kinase activities [222]. As another dual inhibitor of S6K1 and AKT, M2698, which is capable of penetrating the blood–brain barrier, exhibits marked suppression of the activity of S6K1 and AKT1/3 in vitro and in vivo [223, 224]. In a phase I study, M2698 was well tolerated. In combination with tamoxifen or trastuzumab, M2698 manifests antitumor effects on patients with advanced cancer who are unresponsive to multiple standard therapies [225]. Gingerenone A is a natural compound extracted from ginger that could serve as a dual inhibitor of S6K1 and JAK2, as evidenced by a marked reduction in S6K1 phosphorylation. Gingerenone A functions in suppressing cell and tumor growth in mice [226]. Chen et al. also provided evidence that gingerenone A enhances insulin sensitivity and glucose uptake, possibly by inhibiting S6K1-IRS1 negative feedback regulation [227]. Several natural components also show inhibitory effects toward S6K1, which has been well summarized previously [44].

### mTORC1 inhibitors indirectly repress S6K activity

Due to its essential roles in cell physiological processes, aberrations of mTORC1 are frequently implicated in various diseases, including cancers. Thus, targeting mTORC1 represents a powerful apparatus for combating diseases [92]. The first and most well-studied inhibitor of mTORC1 is rapamycin, whose unpleasant water solubility and chemical stability promote the development of rapalogs to induce apoptosis by heterodimerization with FKBP12, suppressing the activation of S6K1 and 4E-BP1 [228]. However, clinical trials for mTORC1 inhibitors are unsatisfactory, possibly due to the release of AKT activation induced by the negative feedback regulation of S6K1 [152, 154]. Therefore, inhibitors exhibiting dual suppression of mTORC1 and mTORC2 have emerged as a promising strategy alone or in combination with other agents. In addition, the active fraction of Streptomyces sp OA293 shows potent inhibition of mTORC1 and AKT activation induced by active mTORC2, which is accompanied by suppression of S6K1 and 4E-BP1 [229]. In addition to rapalogs, ATP analogs also represent another prospective inhibitor of mTOR by competing with ATP binding to mTOR kinase [230]. As an ATP competitive inhibitor, CC-223 has been reported to exhibit tolerable and manageable toxicities when treating patients who had advanced cancer in a phase I study [231]. A subsequent phase II study among well-differentiated neuroendocrine tumor (NET) patients revealed highly durable tumor regression and a remarkable reduction in NET carcinoid symptoms, suggesting that CC-223 is a promising agent [232]. Combination usage of PI3K, AKT, and mTOR inhibitors (AZD8055, GDC0941, and selumetinib) markedly reduces cell proliferation and retards tumor growth in PDX mouse models of ovarian clear cell carcinoma [233].

### PDK1 inhibitors function indirectly in fine-tuning S6K activity

As another key regulator of S6Ks, PDK1 inhibition could lead to suppression of S6Ks. Unlike mTOR inhibitors, the development of PDK1 inhibitors is still in a start-up stage. PDK1 possesses an ATP binding pocket, PDK1-interacting fragment (PIF)-binding pocket, and phosphate-binding pocket, which could be targeted to develop specific inhibitors for PDK1. Of note, SBF1 may serve as a specific allosteric inhibitor of PDK1 anchoring the PIF pocket, as a promising scaffold template for future modulation [234]. Derived from celecoxib, OSU-03012 has emerged as a potent inhibitor of PDK1-AKT signaling by targeting the ATP binding pocket of PDK1 [235]. OSU-03012 has been tested in various cancers, such as pancreatic, esophageal, and endometrial carcinoma cells, and is capable of inducing autophagy and increasing ROS levels and subsequently suppressing cell growth [236]. GSK470 has also been developed as a specific PDK1 inhibitor to suppress cell growth and enhance cell death of multiple myeloma cells, synergized with proteasome inhibitor MG132 [237]. JX06 is also considered a prospective agent for suppressing PDK1 in multiple myeloma cells. In combination with the proteasome inhibitor bortezomib, JX06 has shown synergetic effects on cell death in multiple myeloma cells [238]. Tan et al. reported that BX795 and BX912 might function as PDK1 inhibitors to abolish PDK1-induced c-Myc expression, resulting in cell death and a reduction in tumor sphere formation [239]. Moreover, BX795 synergizes with cisplatin to markedly reduce the epithelial-mesenchymal transition (EMT) in oral squamous cell carcinoma [240]. Dichloroacetat synergizes with the nitric oxide synthase (NOS) inhibitor T1023, reducing tumor growth [241]. It has been illustrated that PHT-427, a dual inhibitor of AKT and PDK1, exhibits promising inhibitory effects in various tumors by binding the PH domains of AKT and PDK1 [242]. Furthermore, efficiently delivering PHT-427 with nanoparticles leads to high oxidative stress levels and enhanced apoptosis in head and neck squamous cell carcinoma [243].

## Conclusion and perspectives

It is generally accepted that S6Ks play oncogenic roles in different cancers, although various isoforms and alternative splices exhibit distinct distributions and contributions in cells and tissues. Specifically, due to the different chromatin locations of genes encoding S6K1 and S6K2, genomic alterations or upstream regulation at the epigenetic and transcriptional levels have shown significant differences, which possibly explains the context-dependent specificity for S6K1 and S6K2. To further investigate the physiological or pathological roles of S6K isoforms, tissue-specific conditional knockout mice for different isoforms are valuable tools and should be generated. Although S6K1 and S6K2 share a similar structure and amino acid sequence, different upstream regulators possibly determine the distinct roles for S6Ks. On the other hand, although posttranslational modifications, such as phosphorylation, have been extensively investigated for S6Ks, other PTMs involved in S6K regulation, such as methylation, ubiquitination, palmitoylation, and their cross talk with phosphorylation and their contributions to S6K kinase activity, warrant further exploration.

S6K1 and S6K2 share most substrates, suggesting potential redundant functions for these S6Ks and highlighting the limitation of specific inhibitors targeting single S6K. Simultaneously, different substrates have also been identified for the S6K isoforms (Fig. 3), indicating distinct functions between S6Ks. This observation will help identify specific inhibitors of S6K1 or S6K2 for precision therapies in a tissue context-dependent manner. Although accumulating substrates have been identified for S6Ks, characterization of novel and specific substrates is also desired to better dissect the S6K functions. Similar to AKT kinase, S6K also phosphorylates its substrates at the R/KxxS/T motif, but interestingly, there are some substrates, such as ERα, H2B, S6, eIF4B, Rictor, and IRS1, which have also been characterized as substrates of AKT or SGK.

As we mentioned above, in addition to controlling cell size by promoting the initiation and elongation processes of protein synthesis, S6Ks also directly contribute to cell proliferation, cell apoptosis, cell metabolic homeostasis, and feedback regulation of their upstream signals by phosphorylating different proteins. These processes integrate to direct S6K functions to mediate mTORC1-dependent or mTORC1-independent tumorigenesis. Thus, it has become promising to target S6K for cancer therapies. However, due to the negative feedback regulation of the PI3K-AKT pathway (Fig. 5A), directly [211, 221] or indirectly [244] targeting S6K will induce elevated AKT kinase activity, which may result in drug resistance. To this end, dual-target inhibitors for S6K and AKT have begun to be developed and display a good response in cancer therapies [219, 225]. Collectively, there is no doubt that S6K plays important roles in tumor cell proliferation, apoptosis, survival, and metastasis by regulating multiple processes, including cell size control and metabolic homeostasis, which highlights a potential strategy to target S6K for cancer therapies in the near future.

## Data Availability

The published article includes all data sets generated/analyzed for this study.

## References

[1] Liu GY, Sabatini DM (2020). mTOR at the nexus of nutrition, growth, ageing and disease. Nat Rev Mol Cell Biol.

[2] Zanconato F, Cordenonsi M, Piccolo S (2019). YAP and TAZ: a signalling hub of the tumour microenvironment. Nat Rev Cancer.

[3] Gan W, Dai X, Dai X, Xie J, Yin S, Zhu J (2020). LATS suppresses mTORC1 activity to directly coordinate Hippo and mTORC1 pathways in growth control. Nat Cell Biol.

[4] Cunningham R, Hansen CG (2022). The Hippo pathway in cancer: YAP/TAZ and TEAD as therapeutic targets in cancer. Clin Sci (Lond).

[5] Valvezan AJ, Manning BD (2019). Molecular logic of mTORC1 signalling as a metabolic rheostat. Nat Metab.

[6] Cormerais Y, Vucetic M, Parks SK, Pouyssegur J (2020). Amino acid transporters are a vital focal point in the control of mTORC1 signaling and cancer. Int J Mol Sci.

[7] Polivka J, Janku F (2014). Molecular targets for cancer therapy in the PI3K/AKT/mTOR pathway. Pharm Ther.

[8] Sancak Y, Bar-Peled L, Zoncu R, Markhard AL, Nada S, Sabatini DM (2010). Ragulator-Rag complex targets mTORC1 to the lysosomal surface and is necessary for its activation by amino acids. Cell.

[9] Xu F, Na L, Li Y, Chen L (2020). Roles of the PI3K/AKT/mTOR signalling pathways in neurodegenerative diseases and tumours. Cell Biosci.

[10] Ruvinsky I, Meyuhas O (2006). Ribosomal protein S6 phosphorylation: from protein synthesis to cell size. Trends Biochem Sci.

[11] Blume-Jensen P, Hunter T (2001). Oncogenic kinase signalling. Nature.

[12] Guo J, Chakraborty AA, Liu P, Gan W, Zheng X, Inuzuka H (2016). pVHL suppresses kinase activity of Akt in a proline-hydroxylation-dependent manner. Science.

[13] Dong C, Wu J, Chen Y, Nie J, Chen C (2021). Activation of PI3K/AKT/mTOR pathway causes drug resistance in breast cancer. Front Pharm.

[14] Copps KD, Hançer NJ, Qiu W, White MF (2016). Serine 302 phosphorylation of mouse insulin receptor substrate 1 (IRS1) is dispensable for normal insulin signaling and feedback regulation by hepatic S6 kinase. J Biol Chem.

[15] Dagon Y, Hur E, Zheng B, Wellenstein K, Cantley LC, Kahn BB (2012). p70S6 kinase phosphorylates AMPK on serine 491 to mediate leptin's effect on food intake. Cell Metab.

[16] Kim K, Pyo S, Um SH (2012). S6 kinase 2 deficiency enhances ketone body production and increases peroxisome proliferator-activated receptor alpha activity in the liver. Hepatology.

[17] Leroux AE, Schulze JO, Biondi RM (2018). AGC kinases, mechanisms of regulation and innovative drug development. Semin Cancer Biol.

[18] Pearce LR, Komander D, Alessi DR (2010). The nuts and bolts of AGC protein kinases. Nat Rev Mol Cell Biol.

[19] Manning G, Whyte DB, Martinez R, Hunter T, Sudarsanam S (2002). The protein kinase complement of the human genome. Science.

[20] Fleuren ED, Zhang L, Wu J, Daly RJ (2016). The kinome 'at large' in cancer. Nat Rev Cancer.

[21] Prêtre V, Wicki A (2018). Inhibition of Akt and other AGC kinases: a target for clinical cancer therapy?. Semin Cancer Biol.

[22] Sridharan S, Basu A (2020). Distinct roles of mTOR targets S6K1 and S6K2 in breast cancer. Int J Mol Sci.

[23] Pardo OE, Seckl MJ (2013). S6K2: the neglected S6 kinase family member. Front Oncol.

[24] Rosner M, Hengstschlager M (2011). Nucleocytoplasmic localization of p70 S6K1, but not of its isoforms p85 and p31, is regulated by TSC2/mTOR. Oncogene.

[25] Grove JR, Banerjee P, Balasubramanyam A, Coffer PJ, Price DJ, Avruch J (1991). Cloning and expression of two human p70 S6 kinase polypeptides differing only at their amino termini. Mol Cell Biol.

[26] Gout I, Minami T, Hara K, Tsujishita Y, Filonenko V, Waterfield MD (1998). Molecular cloning and characterization of a novel p70 S6 kinase, p70 S6 kinase beta containing a proline-rich region. J Biol Chem.

[27] Tavares MR, Pavan IC, Amaral CL, Meneguello L, Luchessi AD, Simabuco FM (2015). The S6K protein family in health and disease. Life Sci.

[28] Zhang J, Guo J, Qin X, Wang B, Zhang L, Wang Y (2018). The p85 isoform of the kinase S6K1 functions as a secreted oncoprotein to facilitate cell migration and tumor growth. Sci Signal.

[29] Karni R, de Stanchina E, Lowe SW, Sinha R, Mu D, Krainer AR (2007). The gene encoding the splicing factor SF2/ASF is a proto-oncogene. Nat Struct Mol Biol.

[30] Ben-Hur V, Denichenko P, Siegfried Z, Maimon A, Krainer A, Davidson B (2013). S6K1 alternative splicing modulates its oncogenic activity and regulates mTORC1. Cell Rep.

[31] Fenton TR, Gout IT (2011). Functions and regulation of the 70kDa ribosomal S6 kinases. Int J Biochem Cell Biol.

[32] Burnett PE, Blackshaw S, Lai MM, Qureshi IA, Burnett AF, Sabatini DM (1998). Neurabin is a synaptic protein linking p70 S6 kinase and the neuronal cytoskeleton. Proc Natl Acad Sci USA.

[33] Magnuson B, Ekim B, Fingar DC (2012). Regulation and function of ribosomal protein S6 kinase (S6K) within mTOR signalling networks. Biochem J.

[34] Aguilar V, Alliouachene S, Sotiropoulos A, Sobering A, Athea Y, Djouadi F (2007). S6 kinase deletion suppresses muscle growth adaptations to nutrient availability by activating AMP kinase. Cell Metab.

[35] Levine ME, Suarez JA, Brandhorst S, Balasubramanian P, Cheng CW, Madia F (2014). Low protein intake is associated with a major reduction in IGF-1, cancer, and overall mortality in the 65 and younger but not older population. Cell Metab.

[36] Selman C, Tullet JM, Wieser D, Irvine E, Lingard SJ, Choudhury AI (2009). Ribosomal protein S6 kinase 1 signaling regulates mammalian life span. Science.

[37] Bae EJ, Xu J, Oh DY, Bandyopadhyay G, Lagakos WS, Keshwani M (2012). Liver-specific p70 S6 kinase depletion protects against hepatic steatosis and systemic insulin resistance. J Biol Chem.

[38] Um SH, Frigerio F, Watanabe M, Picard F, Joaquin M, Sticker M (2004). Absence of S6K1 protects against age- and diet-induced obesity while enhancing insulin sensitivity. Nature.

[39] Querfurth H, Lee HK (2021). Mammalian/mechanistic target of rapamycin (mTOR) complexes in neurodegeneration. Mol Neurodegener.

[40] Ma J, Kala S, Yung S, Chan TM, Cao Y, Jiang Y (2018). Blocking stemness and metastatic properties of ovarian cancer cells by targeting p70(S6K) with dendrimer nanovector-based siRNA delivery. Mol Ther.

[41] Li PD, Zhang WJ, Zhang MY, Yuan LJ, Cha YL, Ying XF (2012). Overexpression of RPS6KB1 predicts worse prognosis in primary HCC patients. Med Oncol.

[42] Amaral CL, Freitas LB, Tamura RE, Tavares MR, Pavan IC, Bajgelman MC (2016). S6Ks isoforms contribute to viability, migration, docetaxel resistance and tumor formation of prostate cancer cells. BMC Cancer.

[43] Pérez-Tenorio G, Karlsson E, Waltersson MA, Olsson B, Holmlund B, Nordenskjöld B (2011). Clinical potential of the mTOR targets S6K1 and S6K2 in breast cancer. Breast Cancer Res Treat.

[44] Ip CK, Wong AS (2012). Exploiting p70 S6 kinase as a target for ovarian cancer. Expert Opin Ther Targets.

[45] Nakamura JL, Garcia E, Pieper RO (2008). S6K1 plays a key role in glial transformation. Cancer Res.

[46] Lu Z, Peng K, Wang N, Liu HM, Hou G (2016). Downregulation of p70S6K enhances cell sensitivity to rapamycin in esophageal squamous cell carcinoma. J Immunol Res.

[47] Du W, Gerald D, Perruzzi CA, Rodriguez-Waitkus P, Enayati L, Krishnan B (2013). Vascular tumors have increased p70 S6-kinase activation and are inhibited by topical rapamycin. Lab Invest.

[48] Yoshida S, Matsumoto K, Arao T, Taniguchi H, Goto I, Hanafusa T (2013). Gene amplification of ribosomal protein S6 kinase-1 and -2 in gastric cancer. Anticancer Res.

[49] Pardo OE, Wellbrock C, Khanzada UK, Aubert M, Arozarena I, Davidson S (2006). FGF-2 protects small cell lung cancer cells from apoptosis through a complex involving PKCepsilon, B-Raf and S6K2. EMBO J.

[50] Sahin F, Kannangai R, Adegbola O, Wang J, Su G, Torbenson M (2004). mTOR and P70 S6 kinase expression in primary liver neoplasms. Clin Cancer Res.

[51] Pende M, Um SH, Mieulet V, Sticker M, Goss VL, Mestan J (2004). S6K1(-/-)/S6K2(-/-) mice exhibit perinatal lethality and rapamycin-sensitive 5'-terminal oligopyrimidine mRNA translation and reveal a mitogen-activated protein kinase-dependent S6 kinase pathway. Mol Cell Biol.

[52] Erikson E, Maller JL (1989). In vivo phosphorylation and activation of ribosomal protein S6 kinases during Xenopus oocyte maturation. J Biol Chem.

[53] Roux PP, Shahbazian D, Vu H, Holz MK, Cohen MS, Taunton J (2007). RAS/ERK signaling promotes site-specific ribosomal protein S6 phosphorylation via RSK and stimulates cap-dependent translation. J Biol Chem.

[54] Catez F, Dalla Venezia N, Marcel V, Zorbas C, Lafontaine DLJ, Diaz JJ (2019). Ribosome biogenesis: An emerging druggable pathway for cancer therapeutics. Biochem Pharm.

[55] Chauvin C, Koka V, Nouschi A, Mieulet V, Hoareau-Aveilla C, Dreazen A (2014). Ribosomal protein S6 kinase activity controls the ribosome biogenesis transcriptional program. Oncogene.

[56] Ghosh J, Kobayashi M, Ramdas B, Chatterjee A, Ma P, Mali RS (2016). S6K1 regulates hematopoietic stem cell self-renewal and leukemia maintenance. J Clin Invest.

[57] Lee S, Roh HS, Song SS, Shin J, Lee J, Bhang DH (2020). Loss of S6K1 but not S6K2 in the tumor microenvironment suppresses tumor growth by attenuating tumor angiogenesis. Transl Oncol.

[58] Pende M, Kozma SC, Jaquet M, Oorschot V, Burcelin R, Le Marchand-Brustel Y (2000). Hypoinsulinaemia, glucose intolerance and diminished beta-cell size in S6K1-deficient mice. Nature.

[59] Ruvinsky I, Sharon N, Lerer T, Cohen H, Stolovich-Rain M, Nir T (2005). Ribosomal protein S6 phosphorylation is a determinant of cell size and glucose homeostasis. Genes Dev.

[60] Irvine EE, Katsouri L, Plattner F, Al-Qassab H, Al-Nackkash R, Bates GP (2019). Genetic deletion of S6k1 does not rescue the phenotypic deficits observed in the R6/2 mouse model of Huntington's disease. Sci Rep.

[61] Caccamo A, Branca C, Talboom JS, Shaw DM, Turner D, Ma L (2015). Reducing ribosomal protein S6 kinase 1 expression improves spatial memory and synaptic plasticity in a mouse model of Alzheimer's disease. J Neurosci.

[62] Chen JK, Chen J, Thomas G, Kozma SC, Harris RC (2009). S6 kinase 1 knockout inhibits uninephrectomy- or diabetes-induced renal hypertrophy. Am J Physiol Ren Physiol.

[63] Ohanna M, Sobering AK, Lapointe T, Lorenzo L, Praud C, Petroulakis E (2005). Atrophy of S6K1(-/-) skeletal muscle cells reveals distinct mTOR effectors for cell cycle and size control. Nat Cell Biol.

[64] Shioi T, McMullen JR, Tarnavski O, Converso K, Sherwood MC, Manning WJ (2003). Rapamycin attenuates load-induced cardiac hypertrophy in mice. Circulation.

[65] Shioi T, Kang PM, Douglas PS, Hampe J, Yballe CM, Lawitts J (2000). The conserved phosphoinositide 3-kinase pathway determines heart size in mice. EMBO J.

[66] McMullen JR, Shioi T, Zhang L, Tarnavski O, Sherwood MC, Dorfman AL (2004). Deletion of ribosomal S6 kinases does not attenuate pathological, physiological, or insulin-like growth factor 1 receptor-phosphoinositide 3-kinase-induced cardiac hypertrophy. Mol Cell Biol.

[67] Cho CS, Kowalsky AH, Lee JH (2020). Pathological consequences of hepatic mTORC1 dysregulation. Genes.

[68] Guertin DA, Stevens DM, Thoreen CC, Burds AA, Kalaany NY, Moffat J (2006). Ablation in mice of the mTORC components raptor, rictor, or mLST8 reveals that mTORC2 is required for signaling to Akt-FOXO and PKCalpha, but not S6K1. Dev Cell.

[69] Han JM, Sahin M (2011). TSC1/TSC2 signaling in the CNS. FEBS Lett.

[70] Qin Z, Zheng H, Zhou L, Ou Y, Huang B, Yan B (2016). Tsc1 deficiency impairs mammary development in mice by suppression of AKT, nuclear ERalpha, and cell-cycle-driving proteins. Sci Rep.

[71] Barlund M, Monni O, Kononen J, Cornelison R, Torhorst J, Sauter G (2000). Multiple genes at 17q23 undergo amplification and overexpression in breast cancer. Cancer Res.

[72] Venkatasubramani JP, Subramanyam P, Pal R, Reddy BK, Srinivasan DJ, Chattarji S (2020). N-terminal variant Asp14Asn of the human p70 S6 Kinase 1 enhances translational signaling causing different effects in developing and mature neuronal cells. Neurobiol Learn Mem.

[73] Maruani DM, Spiegel TN, Harris EN, Shachter AS, Unger HA, Herrero-Gonzalez S (2012). Estrogenic regulation of S6K1 expression creates a positive regulatory loop in control of breast cancer cell proliferation. Oncogene.

[74] Berman AY, Manna S, Schwartz NS, Katz YE, Sun Y, Behrmann CA (2017). ERRalpha regulates the growth of triple-negative breast cancer cells via S6K1-dependent mechanism. Signal Transduct Target Ther.

[75] Song J, Richard S (2015). Sam68 regulates S6K1 alternative splicing during adipogenesis. Mol Cell Biol.

[76] Janaki Ramaiah M, Lavanya A, Honarpisheh M, Zarea M, Bhadra U, Bhadra MP (2014). MiR-15/16 complex targets p70S6 kinase 1 and controls cell proliferation in MDA-MB-231 breast cancer cells. Gene.

[77] Tao T, Li G, Dong Q, Liu D, Liu C, Han D (2014). Loss of SNAIL inhibits cellular growth and metabolism through the miR-128-mediated RPS6KB1/HIF-1α/PKM2 signaling pathway in prostate cancer cells. Tumour Biol.

[78] Cai C, Chen QB, Han ZD, Zhang YQ, He HC, Chen JH (2015). miR-195 inhibits tumor progression by targeting RPS6KB1 in human prostate cancer. Clin Cancer Res.

[79] Yu T, Li J, Yan M, Liu L, Lin H, Zhao F (2015). MicroRNA-193a-3p and -5p suppress the metastasis of human non-small-cell lung cancer by downregulating the ERBB4/PIK3R3/mTOR/S6K2 signaling pathway. Oncogene.

[80] Razaviyan J, Hadavi R, Tavakoli R, Kamani F, Paknejad M, Mohammadi-Yeganeh S (2018). Expression of miRNAs targeting mTOR and S6K1 genes of mTOR signaling pathway including miR-96, miR-557, and miR-3182 in triple-negative breast cancer. Appl Biochem Biotechnol.

[81] Zhong FY, Li J, Wang YM, Chen Y, Song J, Yang Z (2021). MicroRNA-506 modulates insulin resistance in human adipocytes by targeting S6K1 and altering the IRS1/PI3K/AKT insulin signaling pathway. J Bioenerg Biomembr.

[82] Wang L, Sun J, Han J, Ma Z, Pan M, Du Z (2021). MiR-181a promotes spermatogenesis by targeting the S6K1 pathway. Int J Stem Cells.

[83] Liu Y, Zong ZH, Guan X, Wang LL, Zhao Y (2017). The role of long non-coding RNA PCA3 in epithelial ovarian carcinoma tumorigenesis and progression. Gene.

[84] Wang M, Wang L, He X, Zhang J, Zhu Z, Zhang M (2020). lncRNA CCAT2 promotes radiotherapy resistance for human esophageal carcinoma cells via the miR145/p70S6K1 and p53 pathway. Int J Oncol.

[85] Mukhopadhyay NK, Price DJ, Kyriakis JM, Pelech S, Sanghera J, Avruch J (1992). An array of insulin-activated, proline-directed serine/threonine protein kinases phosphorylate the p70 S6 kinase. J Biol Chem.

[86] Zhang J, Gao Z, Ye J (2013). Phosphorylation and degradation of S6K1 (p70S6K1) in response to persistent JNK1 Activation. Biochim Biophys Acta.

[87] Isotani S, Hara K, Tokunaga C, Inoue H, Avruch J, Yonezawa K (1999). Immunopurified mammalian target of rapamycin phosphorylates and activates p70 S6 kinase alpha in vitro. J Biol Chem.

[88] Alessi DR, Kozlowski MT, Weng QP, Morrice N, Avruch J (1998). 3-Phosphoinositide-dependent protein kinase 1 (PDK1) phosphorylates and activates the p70 S6 kinase in vivo and in vitro. Curr Biol.

[89] Dibble CC, Elis W, Menon S, Qin W, Klekota J, Asara JM (2012). TBC1D7 is a third subunit of the TSC1-TSC2 complex upstream of mTORC1. Mol Cell.

[90] Sancak Y, Thoreen CC, Peterson TR, Lindquist RA, Kang SA, Spooner E (2007). PRAS40 is an insulin-regulated inhibitor of the mTORC1 protein kinase. Mol Cell.

[91] Inoki K, Li Y, Xu T, Guan KL (2003). Rheb GTPase is a direct target of TSC2 GAP activity and regulates mTOR signaling. Genes Dev.

[92] Saxton RA, Sabatini DM (2017). mTOR signaling in growth, metabolism, and disease. Cell.

[93] Fonseca BD, Alain T, Finestone LK, Huang BP, Rolfe M, Jiang T (2011). Pharmacological and genetic evaluation of proposed roles of mitogen-activated protein kinase/extracellular signal-regulated kinase kinase (MEK), extracellular signal-regulated kinase (ERK), and p90(RSK) in the control of mTORC1 protein signaling by phorbol esters. J Biol Chem.

[94] Lee DF, Kuo HP, Chen CT, Hsu JM, Chou CK, Wei Y (2007). IKK beta suppression of TSC1 links inflammation and tumor angiogenesis via the mTOR pathway. Cell.

[95] Brugarolas J, Lei K, Hurley RL, Manning BD, Reiling JH, Hafen E (2004). Regulation of mTOR function in response to hypoxia by REDD1 and the TSC1/TSC2 tumor suppressor complex. Genes Dev.

[96] Shin S, Wolgamott L, Yu Y, Blenis J, Yoon SO (2011). Glycogen synthase kinase (GSK)-3 promotes p70 ribosomal protein S6 kinase (p70S6K) activity and cell proliferation. Proc Natl Acad Sci USA.

[97] Zhang HH, Lipovsky AI, Dibble CC, Sahin M, Manning BD (2006). S6K1 regulates GSK3 under conditions of mTOR-dependent feedback inhibition of Akt. Mol Cell.

[98] Inoki K, Ouyang H, Zhu T, Lindvall C, Wang Y, Zhang X (2006). TSC2 integrates Wnt and energy signals via a coordinated phosphorylation by AMPK and GSK3 to regulate cell growth. Cell.

[99] Sancak Y, Peterson TR, Shaul YD, Lindquist RA, Thoreen CC, Bar-Peled L (2008). The Rag GTPases bind raptor and mediate amino acid signaling to mTORC1. Science.

[100] Bar-Peled L, Chantranupong L, Cherniack AD, Chen WW, Ottina KA, Grabiner BC (2013). A tumor suppressor complex with GAP activity for the Rag GTPases that signal amino acid sufficiency to mTORC1. Science.

[101] Riojas RA, Kikani CK, Wang C, Mao X, Zhou L, Langlais PR (2006). Fine tuning PDK1 activity by phosphorylation at Ser163. J Biol Chem.

[102] Yang KJ, Shin S, Piao L, Shin E, Li Y, Park KA (2008). Regulation of 3-phosphoinositide-dependent protein kinase-1 (PDK1) by Src involves tyrosine phosphorylation of PDK1 and Src homology 2 domain binding. J Biol Chem.

[103] Wick MJ, Ramos FJ, Chen H, Quon MJ, Dong LQ, Liu F (2003). Mouse 3-phosphoinositide-dependent protein kinase-1 undergoes dimerization and trans-phosphorylation in the activation loop. J Biol Chem.

[104] Casamayor A, Morrice NA, Alessi DR (1999). Phosphorylation of Ser-241 is essential for the activity of 3-phosphoinositide-dependent protein kinase-1: identification of five sites of phosphorylation in vivo. Biochemical J.

[105] Guo J, Cheng J, Zheng N, Zhang X, Dai X, Zhang L (2021). Copper promotes tumorigenesis by activating the PDK1-AKT oncogenic pathway in a copper transporter 1 dependent manner. Adv Sci.

[106] Arif A, Jia J, Willard B, Li X, Fox PL (2019). Multisite phosphorylation of S6K1 directs a kinase phospho-code that determines substrate selection. Mol Cell.

[107] Lai KO, Liang Z, Fei E, Huang H, Ip NY (2015). Cyclin-dependent Kinase 5 (Cdk5)-dependent Phosphorylation of p70 Ribosomal S6 Kinase 1 (S6K) Is Required for Dendritic Spine Morphogenesis. J Biol Chem.

[108] Valovka T, Verdier F, Cramer R, Zhyvoloup A, Fenton T, Rebholz H (2003). Protein kinase C phosphorylates ribosomal protein S6 kinase betaII and regulates its subcellular localization. Mol Cell Biol.

[109] Panasyuk G, Nemazanyy I, Zhyvoloup A, Bretner M, Litchfield DW, Filonenko V (2006). Nuclear export of S6K1 II is regulated by protein kinase CK2 phosphorylation at Ser-17. J Biol Chem.

[110] Rebholz H, Panasyuk G, Fenton T, Nemazanyy I, Valovka T, Flajolet M (2006). Receptor association and tyrosine phosphorylation of S6 kinases. FEBS J.

[111] Peterson RT, Desai BN, Hardwick JS, Schreiber SL (1999). Protein phosphatase 2A interacts with the 70-kDa S6 kinase and is activated by inhibition of FKBP12-rapamycin associated protein. Proc Natl Acad Sci USA.

[112] Petritsch C, Beug H, Balmain A, Oft M (2000). TGF-beta inhibits p70 S6 kinase via protein phosphatase 2A to induce G(1) arrest. Genes Dev.

[113] Hahn K, Miranda M, Francis VA, Vendrell J, Zorzano A, Teleman AA (2010). PP2A regulatory subunit PP2A-B' counteracts S6K phosphorylation. Cell Metab.

[114] Bettoun DJ, Buck DW, Lu J, Khalifa B, Chin WW, Nagpal S (2002). A vitamin D receptor-Ser/Thr phosphatase-p70 S6 kinase complex and modulation of its enzymatic activities by the ligand. J Biol Chem.

[115] Liu J, Stevens PD, Li X, Schmidt MD, Gao T (2011). PHLPP-mediated dephosphorylation of S6K1 inhibits protein translation and cell growth. Mol Cell Biol.

[116] Wang ML, Panasyuk G, Gwalter J, Nemazanyy I, Fenton T, Filonenko V (2008). Regulation of ribosomal protein S6 kinases by ubiquitination. Biochem Biophys Res Commun.

[117] Panasyuk G, Nemazanyy I, Filonenko V, Gout I (2008). Ribosomal protein S6 kinase 1 interacts with and is ubiquitinated by ubiquitin ligase ROC1. Biochem Biophys Res Commun.

[118] Fenton TR, Gwalter J, Ericsson J, Gout IT (2010). Histone acetyltransferases interact with and acetylate p70 ribosomal S6 kinases in vitro and in vivo. Int J Biochem Cell Biol.

[119] Fenton TR, Gwalter J, Cramer R, Gout IT (2010). S6K1 is acetylated at lysine 516 in response to growth factor stimulation. Biochem Biophys Res Commun.

[120] Igarashi M, Guarente L (2016). mTORC1 and SIRT1 cooperate to foster expansion of gut adult stem cells during calorie restriction. Cell.

[121] Das F, Maity S, Ghosh-Choudhury N, Kasinath BS, Ghosh Choudhury G (2019). Deacetylation of S6 kinase promotes high glucose-induced glomerular mesangial cell hypertrophy and matrix protein accumulation. J Biol Chem.

[122] Yang Y, Li X, Luan HH, Zhang B, Zhang K, Nam JH (2020). OGT suppresses S6K1-mediated macrophage inflammation and metabolic disturbance. Proc Natl Acad Sci USA.

[123] Krieg J, Hofsteenge J, Thomas G (1988). Identification of the 40 S ribosomal protein S6 phosphorylation sites induced by cycloheximide. J Biol Chem.

[124] Puighermanal E, Biever A, Pascoli V, Melser S, Pratlong M, Cutando L (2017). Ribosomal protein S6 phosphorylation is involved in novelty-induced locomotion, synaptic plasticity and mRNA translation. Front Mol Neurosci.

[125] Hutchinson JA, Shanware NP, Chang H, Tibbetts RS (2011). Regulation of ribosomal protein S6 phosphorylation by casein kinase 1 and protein phosphatase 1. J Biol Chem.

[126] Pelletier J, Sonenberg N (2019). The organizing principles of eukaryotic ribosome recruitment. Annu Rev Biochem.

[127] Holz MK, Ballif BA, Gygi SP, Blenis J (2005). mTOR and S6K1 mediate assembly of the translation preinitiation complex through dynamic protein interchange and ordered phosphorylation events. Cell.

[128] Tahmasebi S, Khoutorsky A, Mathews MB, Sonenberg N (2018). Translation deregulation in human disease. Nat Rev Mol Cell Biol.

[129] Ma XM, Blenis J (2009). Molecular mechanisms of mTOR-mediated translational control. Nat Rev Mol Cell Biol.

[130] Raught B, Peiretti F, Gingras AC, Livingstone M, Shahbazian D, Mayeur GL (2004). Phosphorylation of eucaryotic translation initiation factor 4B Ser422 is modulated by S6 kinases. EMBO J.

[131] Yang HS, Jansen AP, Komar AA, Zheng X, Merrick WC, Costes S (2003). The transformation suppressor Pdcd4 is a novel eukaryotic translation initiation factor 4A binding protein that inhibits translation. Mol Cell Biol.

[132] Dorrello NV, Peschiaroli A, Guardavaccaro D, Colburn NH, Sherman NE, Pagano M (2006). S6K1- and betaTRCP-mediated degradation of PDCD4 promotes protein translation and cell growth. Science.

[133] Browne GJ, Proud CG (2002). Regulation of peptide-chain elongation in mammalian cells. Eur J Biochem.

[134] Pavan IC, Yokoo S, Granato DC, Meneguello L, Carnielli CM, Tavares MR (2016). Different interactomes for p70-S6K1 and p54-S6K2 revealed by proteomic analysis. Proteomics.

[135] Sfakianos AP, Mellor LE, Pang YF, Kritsiligkou P, Needs H, Abou-Hamdan H (2018). The mTOR-S6 kinase pathway promotes stress granule assembly. Cell Death Differ.

[136] Richardson CJ, Broenstrup M, Fingar DC, Julich K, Ballif BA, Gygi S (2004). SKAR is a specific target of S6 kinase 1 in cell growth control. Curr Biol.

[137] Ma XM, Yoon SO, Richardson CJ, Julich K, Blenis J (2008). SKAR links pre-mRNA splicing to mTOR/S6K1-mediated enhanced translation efficiency of spliced mRNAs. Cell.

[138] Wilson KF, Fortes P, Singh US, Ohno M, Mattaj IW, Cerione RA (1999). The nuclear cap-binding complex is a novel target of growth factor receptor-coupled signal transduction. J Biol Chem.

[139] Wilson KF, Wu WJ, Cerione RA (2000). Cdc42 stimulates RNA splicing via the S6 kinase and a novel S6 kinase target, the nuclear cap-binding complex. J Biol Chem.

[140] Lee Y, Rio DC (2015). Mechanisms and regulation of alternative pre-mRNA splicing. Annu Rev Biochem.

[141] Lee G, Zheng Y, Cho S, Jang C, England C, Dempsey JM (2017). Post-transcriptional regulation of de novo lipogenesis by mTORC1-S6K1-SRPK2 signaling. Cell.

[142] Darnell JC, Mostovetsky O, Darnell RB (2005). FMRP RNA targets: identification and validation. Genes Brain Behav.

[143] Narayanan U, Nalavadi V, Nakamoto M, Thomas G, Ceman S, Bassell GJ (2008). S6K1 phosphorylates and regulates fragile X mental retardation protein (FMRP) with the neuronal protein synthesis-dependent mammalian target of rapamycin (mTOR) signaling cascade. J Biol Chem.

[144] Liu X, Kumar V, Tsai NP, Auerbach BD (2021). Hyperexcitability and homeostasis in fragile X syndrome. Front Mol Neurosci.

[145] Ismail HM, Hurd PJ, Khalil MI, Kouzarides T, Bannister A, Gout I (2014). S6 kinase 2 is bound to chromatin-nuclear matrix cellular fractions and is able to phosphorylate histone H3 at threonine 45 in vitro and in vivo. J Cell Biochem.

[146] Lee JH, Kang BH, Jang H, Kim TW, Choi J, Kwak S (2015). AKT phosphorylates H3-threonine 45 to facilitate termination of gene transcription in response to DNA damage. Nucleic Acids Res.

[147] Horman SR, Janas MM, Litterst C, Wang B, MacRae IJ, Sever MJ (2013). Akt-mediated phosphorylation of argonaute 2 downregulates cleavage and upregulates translational repression of microRNA targets. Mol Cell.

[148] Paroo Z, Ye X, Chen S, Liu Q (2009). Phosphorylation of the human microRNA-generating complex mediates MAPK/Erk signaling. Cell.

[149] Herbert KM, Pimienta G, DeGregorio SJ, Alexandrov A, Steitz JA (2013). Phosphorylation of DGCR8 increases its intracellular stability and induces a progrowth miRNA profile. Cell Rep.

[150] Warner MJ, Bridge KS, Hewitson JP, Hodgkinson MR, Heyam A, Massa BC (2016). S6K2-mediated regulation of TRBP as a determinant of miRNA expression in human primary lymphatic endothelial cells. Nucleic Acids Res.

[151] O'Reilly KE, Rojo F, She QB, Solit D, Mills GB, Smith D (2006). mTOR inhibition induces upstream receptor tyrosine kinase signaling and activates Akt. Cancer Res.

[152] Hoxhaj G, Manning BD (2020). The PI3K-AKT network at the interface of oncogenic signalling and cancer metabolism. Nat Rev Cancer.

[153] Harrington LS, Findlay GM, Gray A, Tolkacheva T, Wigfield S, Rebholz H (2004). The TSC1-2 tumor suppressor controls insulin-PI3K signaling via regulation of IRS proteins. J Cell Biol.

[154] Zhang J, Gao Z, Yin J, Quon MJ, Ye J (2008). S6K directly phosphorylates IRS-1 on Ser-270 to promote insulin resistance in response to TNF-(alpha) signaling through IKK2. J Biol Chem.

[155] Tremblay F, Brule S, Um SH, Li Y, Masuda K, Roden M (2007). Identification of IRS-1 Ser-1101 as a target of S6K1 in nutrient- and obesity-induced insulin resistance. Proc Natl Acad Sci USA.

[156] Holz MK, Blenis J (2005). Identification of S6 kinase 1 as a novel mammalian target of rapamycin (mTOR)-phosphorylating kinase. J Biol Chem.

[157] Chiang GG, Abraham RT (2005). Phosphorylation of mammalian target of rapamycin (mTOR) at Ser-2448 is mediated by p70S6 kinase. J Biol Chem.

[158] Liu P, Gan W, Inuzuka H, Lazorchak AS, Gao D, Arojo O (2013). Sin1 phosphorylation impairs mTORC2 complex integrity and inhibits downstream Akt signalling to suppress tumorigenesis. Nat Cell Biol.

[159] Gao D, Wan L, Inuzuka H, Berg AH, Tseng A, Zhai B (2010). Rictor forms a complex with Cullin-1 to promote SGK1 ubiquitination and destruction. Mol Cell.

[160] Gao D, Wan L, Wei W (2010). Phosphorylation of Rictor at Thr1135 impairs the Rictor/Cullin-1 complex to ubiquitinate SGK1. Protein Cell.

[161] Julien LA, Carriere A, Moreau J, Roux PP (2010). mTORC1-activated S6K1 phosphorylates Rictor on threonine 1135 and regulates mTORC2 signaling. Mol Cell Biol.

[162] Jiang Q, Zhang X, Dai X, Han S, Wu X, Wang L (2022). S6K1-mediated phosphorylation of PDK1 impairs AKT kinase activity and oncogenic functions. Nat Commun.

[163] Harada H, Andersen JS, Mann M, Terada N, Korsmeyer SJ (2001). p70S6 kinase signals cell survival as well as growth, inactivating the pro-apoptotic molecule BAD. Proc Natl Acad Sci USA.

[164] Djouder N, Metzler SC, Schmidt A, Wirbelauer C, Gstaiger M, Aebersold R (2007). S6K1-mediated disassembly of mitochondrial URI/PP1gamma complexes activates a negative feedback program that counters S6K1 survival signaling. Mol Cell.

[165] Vogelstein B, Lane D, Levine AJ (2000). Surfing the p53 network. Nature.

[166] Kubbutat MH, Jones SN, Vousden KH (1997). Regulation of p53 stability by Mdm2. Nature.

[167] Kruse JP, Gu W (2009). Modes of p53 regulation. Cell.

[168] Lai KP, Leong WF, Chau JF, Jia D, Zeng L, Liu H (2010). S6K1 is a multifaceted regulator of Mdm2 that connects nutrient status and DNA damage response. EMBO J.

[169] Xie X, Hu H, Tong X, Li L, Liu X, Chen M (2018). The mTOR-S6K pathway links growth signalling to DNA damage response by targeting RNF168. Nat Cell Biol.

[170] Liwak U, Thakor N, Jordan LE, Roy R, Lewis SM, Pardo OE (2012). Tumor suppressor PDCD4 represses internal ribosome entry site-mediated translation of antiapoptotic proteins and is regulated by S6 kinase 2. Mol Cell Biol.

[171] Dreyfuss G, Kim VN, Kataoka N (2002). Messenger-RNA-binding proteins and the messages they carry. Nat Rev Mol Cell Biol.

[172] Roy R, Durie D, Li H, Liu BQ, Skehel JM, Mauri F (2014). hnRNPA1 couples nuclear export and translation of specific mRNAs downstream of FGF-2/S6K2 signalling. Nucleic Acids Res.

[173] Sun Y, Luo M, Chang G, Ren W, Wu K, Li X (2017). Phosphorylation of Ser6 in hnRNPA1 by S6K2 regulates glucose metabolism and cell growth in colorectal cancer. Oncol Lett.

[174] Minokoshi Y, Alquier T, Furukawa N, Kim YB, Lee A, Xue B (2004). AMP-kinase regulates food intake by responding to hormonal and nutrient signals in the hypothalamus. Nature.

[175] Arif A, Chatterjee P, Moodt RA, Fox PL (2012). Heterotrimeric GAIT complex drives transcript-selective translation inhibition in murine macrophages. Mol Cell Biol.

[176] Kang J, Kim T, Ko YG, Rho SB, Park SG, Kim MJ (2000). Heat shock protein 90 mediates protein-protein interactions between human aminoacyl-tRNA synthetases. J Biol Chem.

[177] Hue L, Rider MH (1987). Role of fructose 2,6-bisphosphate in the control of glycolysis in mammalian tissues. Biochem J.

[178] Deprez J, Vertommen D, Alessi DR, Hue L, Rider MH (1997). Phosphorylation and activation of heart 6-phosphofructo-2-kinase by protein kinase B and other protein kinases of the insulin signaling cascades. J Biol Chem.

[179] Ros S, Schulze A (2013). Balancing glycolytic flux: the role of 6-phosphofructo-2-kinase/fructose 2,6-bisphosphatases in cancer metabolism. Cancer Metab.

[180] Desvergne B, Wahli W (1999). Peroxisome proliferator-activated receptors: nuclear control of metabolism. Endocr Rev.

[181] Yi SA, Um SH, Lee J, Yoo JH, Bang SY, Park EK (2016). S6K1 phosphorylation of H2B mediates EZH2 trimethylation of H3: a determinant of early adipogenesis. Mol Cell.

[182] Yi SA, Lee J, Park JW, Han J, Lee MG, Nam KH (2018). S6K1 controls epigenetic plasticity for the expression of pancreatic alpha/beta cell marker genes. J Cell Biochem.

[183] Laplante M, Sabatini DM (2012). mTOR signaling in growth control and disease. Cell.

[184] Ben-Sahra I, Howell JJ, Asara JM, Manning BD (2013). Stimulation of de novo pyrimidine synthesis by growth signaling through mTOR and S6K1. Science.

[185] Abe Y, Tanaka N (2020). Fine-tuning of GLI activity through arginine methylation: its mechanisms and function. Cells.

[186] Wang Y, Ding Q, Yen CJ, Xia W, Izzo JG, Lang JY (2012). The crosstalk of mTOR/S6K1 and Hedgehog pathways. Cancer Cell.

[187] Yan B, Zhang Z, Jin D, Cai C, Jia C, Liu W (2016). mTORC1 regulates PTHrP to coordinate chondrocyte growth, proliferation and differentiation. Nat Commun.

[188] Yamnik RL, Digilova A, Davis DC, Brodt ZN, Murphy CJ, Holz MK (2009). S6 kinase 1 regulates estrogen receptor alpha in control of breast cancer cell proliferation. J Biol Chem.

[189] Yamnik RL, Holz MK (2010). mTOR/S6K1 and MAPK/RSK signaling pathways coordinately regulate estrogen receptor alpha serine 167 phosphorylation. FEBS Lett.

[190] Becker MA, Ibrahim YH, Cui X, Lee AV, Yee D (2011). The IGF pathway regulates ERα through a S6K1-dependent mechanism in breast cancer cells. Mol Endocrinol.

[191] Citro S, Miccolo C, Meloni L, Chiocca S (2015). PI3K/mTOR mediate mitogen-dependent HDAC1 phosphorylation in breast cancer: a novel regulation of estrogen receptor expression. J Mol Cell Biol.

[192] Dang CV (2012). MYC on the path to cancer. Cell.

[193] Huang Y, Hu K, Zhang S, Dong X, Yin Z, Meng R (2018). S6K1 phosphorylation-dependent degradation of Mxi1 by beta-Trcp ubiquitin ligase promotes Myc activation and radioresistance in lung cancer. Theranostics.

[194] Kim SY, Baik KH, Baek KH, Chah KH, Kim KA, Moon G (2014). S6K1 negatively regulates TAK1 activity in the toll-like receptor signaling pathway. Mol Cell Biol.

[195] Shin JH, Min SH, Kim SJ, Kim YI, Park J, Lee HK (2013). TAK1 regulates autophagic cell death by suppressing the phosphorylation of p70 S6 kinase 1. Sci Rep.

[196] Ouyang W, Kolls JK, Zheng Y (2008). The biological functions of T helper 17 cell effector cytokines in inflammation. Immunity.

[197] Zhu J, Davidson TS, Wei G, Jankovic D, Cui K, Schones DE (2009). Down-regulation of Gfi-1 expression by TGF-beta is important for differentiation of Th17 and CD103+ inducible regulatory T cells. J Exp Med.

[198] Kurebayashi Y, Nagai S, Ikejiri A, Ohtani M, Ichiyama K, Baba Y (2012). PI3K-Akt-mTORC1-S6K1/2 axis controls Th17 differentiation by regulating Gfi1 expression and nuclear translocation of RORgamma. Cell Rep.

[199] Xu WD, Zhang YJ, Wang W, Li R, Pan HF, Ye DQ (2012). Role of CREM in systemic lupus erythematosus. Cell Immunol.

[200] de Groot RP, Ballou LM, Sassone-Corsi P (1994). Positive regulation of the cAMP-responsive activator CREM by the p70 S6 kinase: an alternative route to mitogen-induced gene expression. Cell.

[201] Abe Y, Yoon SO, Kubota K, Mendoza MC, Gygi SP, Blenis J (2009). p90 ribosomal S6 kinase and p70 ribosomal S6 kinase link phosphorylation of the eukaryotic chaperonin containing TCP-1 to growth factor, insulin, and nutrient signaling. J Biol Chem.

[202] Barilari M, Bonfils G, Treins C, Koka V, De Villeneuve D, Fabrega S (2017). ZRF1 is a novel S6 kinase substrate that drives the senescence programme. EMBO J.

[203] Ip CK, Cheung AN, Ngan HY, Wong AS (2011). p70 S6 kinase in the control of actin cytoskeleton dynamics and directed migration of ovarian cancer cells. Oncogene.

[204] Ismail HM, Myronova O, Tsuchiya Y, Niewiarowski A, Tsaneva I, Gout I (2013). Identification of the general transcription factor Yin Yang 1 as a novel and specific binding partner for S6 kinase 2. Cell Signal.

[205] Pearce LR, Alton GR, Richter DT, Kath JC, Lingardo L, Chapman J (2010). Characterization of PF-4708671, a novel and highly specific inhibitor of p70 ribosomal S6 kinase (S6K1). Biochem J.

[206] Grasso S, Tristante E, Saceda M, Carbonell P, Mayor-López L, Carballo-Santana M (2014). Resistance to Selumetinib (AZD6244) in colorectal cancer cell lines is mediated by p70S6K and RPS6 activation. Neoplasia.

[207] Qiu ZX, Sun RF, Mo XM, Li WM (2016). The p70S6K specific inhibitor PF-4708671 impedes non-small cell lung cancer growth. PLoS ONE.

[208] Al-Ali H, Ding Y, Slepak T, Wu W, Sun Y, Martinez Y (2017). The mTOR substrate S6 kinase 1 (S6K1) is a negative regulator of axon regeneration and a potential drug target for central nervous system injury. J Neurosci.

[209] Shum M, Bellmann K, St-Pierre P, Marette A (2016). Pharmacological inhibition of S6K1 increases glucose metabolism and Akt signalling in vitro and in diet-induced obese mice. Diabetologia.

[210] Gerstenecker S, Haarer L, Schroder M, Kudolo M, Schwalm MP, Wydra V (2021). Discovery of a potent and highly isoform-selective inhibitor of the neglected ribosomal protein S6 kinase beta 2 (S6K2). Cancers.

[211] Doscas ME, Williamson AJ, Usha L, Bogachkov Y, Rao GS, Xiao F (2014). Inhibition of p70 S6 kinase (S6K1) activity by A77 1726 and its effect on cell proliferation and cell cycle progress. Neoplasia.

[212] Segatto I, Massarut S, Boyle R, Baldassarre G, Walker D, Belletti B (2016). Preclinical validation of a novel compound targeting p70S6 kinase in breast cancer. Aging.

[213] Qin J, Rajaratnam R, Feng L, Salami J, Barber-Rotenberg JS, Domsic J (2015). Development of organometallic S6K1 inhibitors. J Med Chem.

[214] Tolcher A, Goldman J, Patnaik A, Papadopoulos KP, Westwood P, Kelly CS (2014). A phase I trial of LY2584702 tosylate, a p70 S6 kinase inhibitor, in patients with advanced solid tumours. Eur J Cancer.

[215] Hollebecque A, Houede N, Cohen EE, Massard C, Italiano A, Westwood P (2014). A phase Ib trial of LY2584702 tosylate, a p70 S6 inhibitor, in combination with erlotinib or everolimus in patients with solid tumours. Eur J Cancer.

[216] Leohr JK, Luffer-Atlas D, Luo MJ, DeBrota DJ, Green C, Mabry TE (2018). Serum lipid and protein changes in healthy dyslipidemic subjects given a selective inhibitor of p70 S6 kinase-1. J Clin Pharm.

[217] Azaro A, Rodon J, Calles A, Brana I, Hidalgo M, Lopez-Casas PP (2015). A first-in-human phase I trial of LY2780301, a dual p70 S6 kinase and Akt Inhibitor, in patients with advanced or metastatic cancer. Invest N. Drugs.

[218] Angevin E, Cassier PA, Italiano A, Goncalves A, Gazzah A, Terret C (2017). Safety, tolerability and antitumour activity of LY2780301 (p70S6K/AKT inhibitor) in combination with gemcitabine in molecularly selected patients with advanced or metastatic cancer: a phase IB dose escalation study. Eur J Cancer.

[219] Vicier C, Sfumato P, Isambert N, Dalenc F, Robert M, Levy C (2021). TAKTIC: a prospective, multicentre, uncontrolled, phase IB/II study of LY2780301, a p70S6K/AKT inhibitor, in combination with weekly paclitaxel in HER2-negative advanced breast cancer patients. Eur J Cancer.

[220] Li S, Brown MS, Goldstein JL (2010). Bifurcation of insulin signaling pathway in rat liver: mTORC1 required for stimulation of lipogenesis, but not inhibition of gluconeogenesis. Proc Natl Acad Sci USA.

[221] Li C, Ma B, Chen J, Jeong Y, Xu X (2020). Astaxanthin inhibits p70 S6 kinase 1 activity to sensitize insulin signaling. Mar Drugs.

[222] Dar AC, Das TK, Shokat KM, Cagan RL (2012). Chemical genetic discovery of targets and anti-targets for cancer polypharmacology. Nature.

[223] Machl A, Wilker EW, Tian H, Liu XH, Schroeder P, Clark A (2016). M2698 is a potent dual-inhibitor of p70S6K and Akt that affects tumor growth in mouse models of cancer and crosses the blood-brain barrier. Am J Cancer Res.

[224] DeSelm L, Huck B, Lan R, Neagu C, Potnick J, Xiao Y (2021). Identification of clinical candidate M2698, a dual p70S6K and Akt inhibitor, for treatment of PAM pathway-altered cancers. J Med Chem.

[225] Tsimberidou AM, Shaw JV, Juric D, Verschraegen C, Weise AM, Sarantopoulos J (2021). Phase 1 study of M2698, a p70S6K/AKT dual inhibitor, in patients with advanced cancer. J Hematol Oncol.

[226] Byun S, Lim S, Mun JY, Kim KH, Ramadhar TR, Farrand L (2015). Identification of a dual inhibitor of janus kinase 2 (JAK2) and p70 ribosomal S6 kinase1 (S6K1) pathways. J Biol Chem.

[227] Chen J, Sun J, Prinz RA, Li Y, Xu X (2018). Gingerenone A sensitizes the insulin receptor and increases glucose uptake by inhibiting the activity of p70 S6 kinase. Mol Nutr Food Res.

[228] Chen Y, Zhou X (2020). Research progress of mTOR inhibitors. Eur J Med Chem.

[229] Dan VM, Muralikrishnan B, Sanawar R, Vinodh JS, Burkul BB, Srinivas KP (2018). Streptomyces sp metabolite(s) promotes Bax mediated intrinsic apoptosis and autophagy involving inhibition of mTOR pathway in cervical cancer cell lines. Sci Rep.

[230] Benjamin D, Colombi M, Moroni C, Hall MN (2011). Rapamycin passes the torch: a new generation of mTOR inhibitors. Nat Rev Drug Disco.

[231] Bendell JC, Kelley RK, Shih KC, Grabowsky JA, Bergsland E, Jones S (2015). A phase I dose-escalation study to assess safety, tolerability, pharmacokinetics, and preliminary efficacy of the dual mTORC1/mTORC2 kinase inhibitor CC-223 in patients with advanced solid tumors or multiple myeloma. Cancer.

[232] Wolin E, Mita A, Mahipal A, Meyer T, Bendell J, Nemunaitis J (2019). A phase 2 study of an oral mTORC1/mTORC2 kinase inhibitor (CC-223) for non-pancreatic neuroendocrine tumors with or without carcinoid symptoms. PLoS ONE.

[233] Caumanns JJ, van Wijngaarden A, Kol A, Meersma GJ, Jalving M, Bernards R (2019). Low-dose triple drug combination targeting the PI3K/AKT/mTOR pathway and the MAPK pathway is an effective approach in ovarian clear cell carcinoma. Cancer Lett.

[234] Liu W, Li P, Mei Y (2019). Discovery of SBF1 as an allosteric inhibitor targeting the PIF-pocket of 3-phosphoinositide-dependent protein kinase-1. J Mol Model.

[235] Zhu J, Huang JW, Tseng PH, Yang YT, Fowble J, Shiau CW (2004). From the cyclooxygenase-2 inhibitor celecoxib to a novel class of 3-phosphoinositide-dependent protein kinase-1 inhibitors. Cancer Res.

[236] Ding L, Ren C, Yang L, Wu Z, Li F, Jiang D (2021). OSU-03012 disrupts Akt signaling and prevents endometrial carcinoma progression in vitro and in vivo. Drug Des Devel Ther.

[237] Zhang J, Yang C, Zhou F, Chen X (2018). PDK1 inhibitor GSK2334470 synergizes with proteasome inhibitor MG132 in multiple myeloma cells by inhibiting full AKT activity and increasing nuclear accumulation of the PTEN protein. Oncol Rep.

[238] Kawano Y, Sasano T, Arima Y, Kushima S, Tsujita K, Matsuoka M (2022). A novel PDK1 inhibitor, JX06, inhibits glycolysis and induces apoptosis in multiple myeloma cells. Biochem Biophys Res Commun.

[239] Tan J, Li Z, Lee PL, Guan P, Aau MY, Lee ST (2013). PDK1 signaling toward PLK1-MYC activation confers oncogenic transformation, tumor-initiating cell activation, and resistance to mTOR-targeted therapy. Cancer Disco.

[240] Pai S, Yadav VK, Kuo KT, Pikatan NW, Lin CS, Chien MH (2021). PDK1 inhibitor BX795 improves cisplatin and radio-efficacy in oral squamous cell carcinoma by downregulating the Pdk1/cd47/akt-mediated glycolysis signaling pathway. Int J Mol Sci.

[241] Filimonova MV, Podosinnikova TS, Samsonova AS, Makarchuk VM, Shevchenko LI, Filimonov AS (2019). Comparison of antitumor effects of combined and separate treatment with NO synthase inhibitor T1023 and PDK1 inhibitor dichloroacetate. Bull Exp Biol Med.

[242] Meuillet EJ, Zuohe S, Lemos R, Ihle N, Kingston J, Watkins R (2010). Molecular pharmacology and antitumor activity of PHT-427, a novel Akt/phosphatidylinositide-dependent protein kinase 1 pleckstrin homology domain inhibitor. Mol Cancer Ther.

[243] Yanes-Diaz J, Palao-Suay R, Aguilar MR, Riestra-Ayora JI, Ferruelo-Alonso A, Rojo Del Olmo L (2021). Antitumor activity of nanoparticles loaded with PHT-427, a Novel AKT/PDK1 inhibitor, for the treatment of head and neck squamous cell carcinoma. Pharmaceutics.

[244] Cheaib B, Auguste A, Leary A (2015). The PI3K/Akt/mTOR pathway in ovarian cancer: therapeutic opportunities and challenges. Chin J Cancer.

